# Transcriptome-Wide *N*^6^-Methyladenosine (m^6^A) Methylation Analyses in a Compatible Wheat–*Puccinia striiformis* f. sp. *tritici* Interaction

**DOI:** 10.3390/plants13070982

**Published:** 2024-03-29

**Authors:** Elif Naz Cerav, Nan Wu, Mahinur S. Akkaya

**Affiliations:** School of Bioengineering, Dalian University of Technology, No. 2 Linggong Road, Dalian 116024, China; elifnazcerav@gmail.com (E.N.C.); wu_nan@mail.dlut.edu.cn (N.W.)

**Keywords:** wheat, *Puccinia striiformis* f. sp. *tritici*, RNA-seq, MeRIP-seq, m^6^A RNA methylation, gene expression, post-transcriptional modification, plant–pathogen interaction, photosynthesis

## Abstract

*N*^6^-methyladenosine (m^6^A) is a prevalent internal modification in eukaryotic mRNA, tRNA, miRNA, and long non-coding RNA. It is also known for its role in plant responses to biotic and abiotic stresses. However, a comprehensive m^6^A transcriptome-wide map for *Puccinia striiformis* f. sp. *tritici* (*Pst*) infections in wheat (*Triticum aestivum*) is currently unavailable. Our study is the first to profile m^6^A modifications in wheat infected with a virulent *Pst* race. Analysis of RNA-seq and MeRIP-seq data revealed that the majority of differentially expressed genes are up-regulated and hyper-methylated. Some of these genes are enriched in the plant–pathogen interaction pathway. Notably, genes related to photosynthesis showed significant down-regulation and hypo-methylation, suggesting a potential mechanism facilitating successful *Pst* invasion by impairing photosynthetic function. The crucial genes, epitomizing the core molecular constituents that fortify plants against pathogenic assaults, were detected with varying expression and methylation levels, together with a newly identified methylation motif. Additionally, m^6^A regulator genes were also influenced by m^6^A modification, and their expression patterns varied at different time points of post-inoculation, with lower expression at early stages of infection. This study provides insights into the role of m^6^A modification regulation in wheat’s response to *Pst* infection, establishing a foundation for understanding the potential function of m^6^A RNA methylation in plant resistance or susceptibility to pathogens.

## 1. Introduction

Many biological processes are controlled by RNA molecules, which play a vital role in transferring genetic information. To perform specific molecular functions, RNA transcripts can be chemically modified. Based on the MODOMICS database (https://iimcb.genesilico.pl/modomics/ (accessed on 1 October 2023)), there have been more than 180 RNA chemical modification discoveries to date, mainly in transfer RNAs (tRNAs) and ribosomal RNAs (rRNAs), where they play important roles in RNA function [1]. The most common post-transcriptional modifications to RNA are the following: *N*^6^-methyladenosine (m^6^A), 5-methylcytosine (m^5^C), *N*^1^-methyladenosine (m^1^A), pseudouridine (Ψ), inosine (I), and *N*^6^,2′-O-dimethyladenosine (m^6^Am) [2].

In eukaryotic mRNA, m^6^A is the most abundant methylation type which is also found in bacteria and RNA viruses [3]. The m^6^A RNA modification is a preserved regulatory system across highly diverse organisms; the system encompasses the methyltransferase complexes known as “writers” that add methyl groups to mRNA, demethylases termed “erasers” that remove these modifications for dynamic regulation, and m^6^A-binding proteins or “readers” which recognize and interact with methylated RNA sites, collectively playing a crucial role in mRNA function by impacting processes such as stability, splicing, localization, and translation, thereby illustrating the evolutionary significance of m^6^A modifications [4,5]. There are several m^6^A methyltransferases, called m^6^A writers, including mRNA adenosine methylases (MTA and MTB), VIRILIZER (VIR), FK506-binding protein 12 (FKBP12)-interacting protein 37 (FIB37), and the E3 ubiquitin ligase HAKAI [6]. In addition, m^6^A demethylases or erasers, such as the alkylated DNA repair proteins AlkB homologs (e.g., AlkBH4b, AlkBH8, AlkBH9B, AlkBH10B, AlkBH11B) [7,8] and fat mass and obesity-associated protein (FTO) [9], can dynamically modulate m^6^A modification. Moreover, there is a series of m^6^A-binding proteins for recognizing m^6^A-containing mRNAs named m^6^A readers like the EVOLUTIONARILY CONSERVED C-TERMINAL REGION (ECT) family (e.g., ECT2, ECT3, ECT4). It has been demonstrated that ECT2 is involved in mRNA stability [10]. YT521-B homology (YTH) family proteins, whose YTH domain can recognize and bind m^6^A-containing RNA, are the main readers of m^6^A [11]. The m^6^A modification very much plays a pivotal role in regulating plant transcription and gene expression, growth, and development [12].

The most canonical consensus motif of the m^6^A modification site is RRACH (R = G or A; H = U, A or C) [13], which accounts for more than 90% of the m^6^A sites in wheat mRNAs [14]. Numerous studies in plants also identified this dominant consensus motif [15,16,17]. Alternative motifs of GGAU and URUAY (Y = C or U) were also found to be enriched in plant species by epitranscriptome analysis [18,19]. The distribution of m^6^A modification within the mRNA is not uniform and is mainly enriched close to the stop codon and 3’ untranslated regions (3’ UTRs) [20]. Despite preliminary research on m^6^A methylation in the plant response to abiotic stress [21,22,23], biotic stress has received far less attention. To highlight a few studies, in *Arabidopsis thaliana*, AtALKBH9B demethylase was reported to erase m^6^A from RNAs of alfalfa mosaic virus (AMV), and its interaction with the CP (coat protein) of AMV resulted in successful viral infection. The suppression of AtALKBH9B led to the hyper-methylation of AMV RNAs and attenuated AMV infectivity, whereas the infection of cucumber mosaic virus (CMV) was unaffected, which might correspond to the absence of interaction between AtALKBH9B and the CMV CP [24]. The mRNAs of certain genes of pear (*Pyrus bretschneideri*) involved in defense mechanisms were m^6^A-modified to enhance the abundance of defense-related transcripts against the inoculation of fire blight pathogen *Erwinia amylovora* [25].

The plant defense regulation mechanism against pathogen assaults has been studied over the years. Many genes were identified based on the changes in expression levels, and the biological and biochemical roles were inferred. The involvement of NBS-LRR genes in plant–pathogen interactions, MAPK signaling, and the biosynthesis of secondary metabolites have been highlighted, and more detailed information is accumulating [26]. Similarly, the pathogenesis-related protein 1 (PR1), along with calcium-dependent protein kinases and calmodulin genes, play crucial roles in plant–pathogen interactions, in signal transduction. The biosynthesis pathways of phenylpropanoids and flavonoids have also been demonstrated [27]. In a study, Wang et al., 2016, emphasized the significance of mitogen-activated protein (MAP) kinase genes in the biosynthesis of phenylpropanoids, flavonoids, stilbenoids, and defense-related pathways [28]. 3-ketoacyl-CoA synthase genes are vital in transcription regulation, signal transduction, plant–pathogen interactions, and MAPK signaling pathways [29]. Additionally, WRKY transcription factor genes, as reported by Chen et al., 2014, are key players in carbohydrate metabolism, fatty acid metabolism, oxidative phosphorylation, and defense and signal transduction pathways [30]. It has also been reported that respiratory burst oxidase genes are involved in the regulation of transcription, transport of organic acids and metal cations, carbohydrate metabolism, and photosynthesis-antenna proteins [31]. Heat shock protein genes were shown to be critical in transcription regulation, metabolism, and signal transduction in stress and defense-related pathways [32]. In interactions between plants and pathogens, the significance of cysteine protease genes in the biosynthesis of phenylpropanoids and defense mechanisms was explored [33] through the extensive identification and characterization of the differential expression during biotic and abiotic stresses [34]. 

One of the most harmful diseases to affect wheat (*Triticum aestivum*) worldwide is known as stripe rust or yellow rust disease and is brought on by *Puccinia striiformis* f. sp. *tritici* (*Pst*). *Pst* leads to a substantial decrease in grain quality and in yield [35]. For long-term field resistance, one aspect is to thoroughly understand the molecular-level interaction mechanisms between wheat and *Pst*, elucidating how wheat responds to stripe rust infection at the molecular level. This study, from the perspective of m^6^A RNA methylation, combined RNA-seq (RNA sequencing) and MeRIP-seq (methylated RNA immunoprecipitation sequencing) analyses to understand the changes in gene expression levels and m^6^A modifications in wheat–*Pst* compatible interaction. Expression level changes in m^6^A regulators of wheat at various time points were also investigated, exploring how m^6^A-mediated post-transcriptional regulation affects the *Pst*-infected wheat, emphasizing a further complexity to the interactions in the pathosystem. We identified the genes going through m^6^A methylation transcriptional regulations in many of the above pathways, introducing an additional level of understanding to the changes in the expression levels. 

## 2. Results

### 2.1. Transcriptome Profile of Wheat under Compatible Pst Interactions

**Figure 1:**
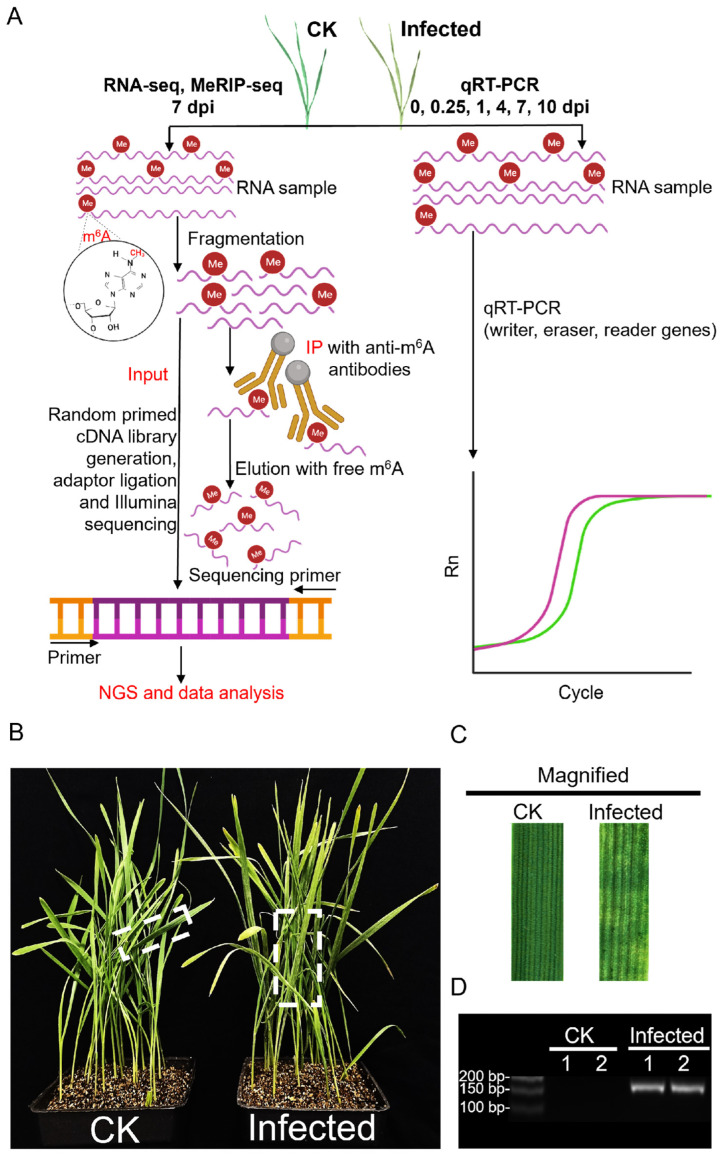
An overview of the experimental setup in wheat after *Pst* infection, integrating MeRIP-seq, RNA-seq, and qRT-PCR with phenotype analysis. (**A**) Schematic of the experimental workflow for MeRIP-seq, RNA-seq, and qRT-PCR in CK and *Pst*-infected wheat leaves. This diagram illustrates the step-by-step procedure adopted for analyzing both mock inoculation wheat leaves (CK) and *Pst*-infected wheat leaves (infected) at 7 dpi using NGS technology. The workflow highlights the incorporation of the MeRIP technique to selectively isolate m^6^A-modified RNA fractions for subsequent sequencing. (**B**) The phenotype of CK and infected wheat seedlings with *Pst* infection at 7 dpi. The CK wheat leaves showed no visible symptoms, but the infected wheat leaves showed chlorotic patches and blotches. (**C**) The magnification of CK and infected wheat leaves in the dotted box in (**B**). (**D**) sqRT-PCR assay of *PsEF1* in CK and infected wheat leaves at 7 dpi.

To understand how the wheat transcriptome responds to attack by *Puccinia striiformis* f. sp. *tritici* (*Pst)* and how it is modified in such an interaction, this study used the Chinese yellow rust race, CYR32 (a virulent *Pst* race on many wheat cultivars in China [36]), to inoculate Avocet/Yr7, creating a compatible interaction model. The total RNA extracted from 7 dpi inoculated and CK samples were utilized for next-generation sequencing (NGS) as input samples, followed by an in-depth analysis of the sequencing data (Figure 1A). The phenotype of inoculated wheat leaves compared to CK showed slight chlorosis with the infection sites exhibiting necrotic, chlorotic flecks and blotches and, only in a few, sporadic sporulation (Figure 1B,C). The successful infection was verified with the expression, and the accumulation of the protein synthesis elongation factor 1 gene, *PsEF1*, belonging to *Pst* was verified with consistency across two biological replicates (Figure 1D and Figure S1A).

**Figure 2:**
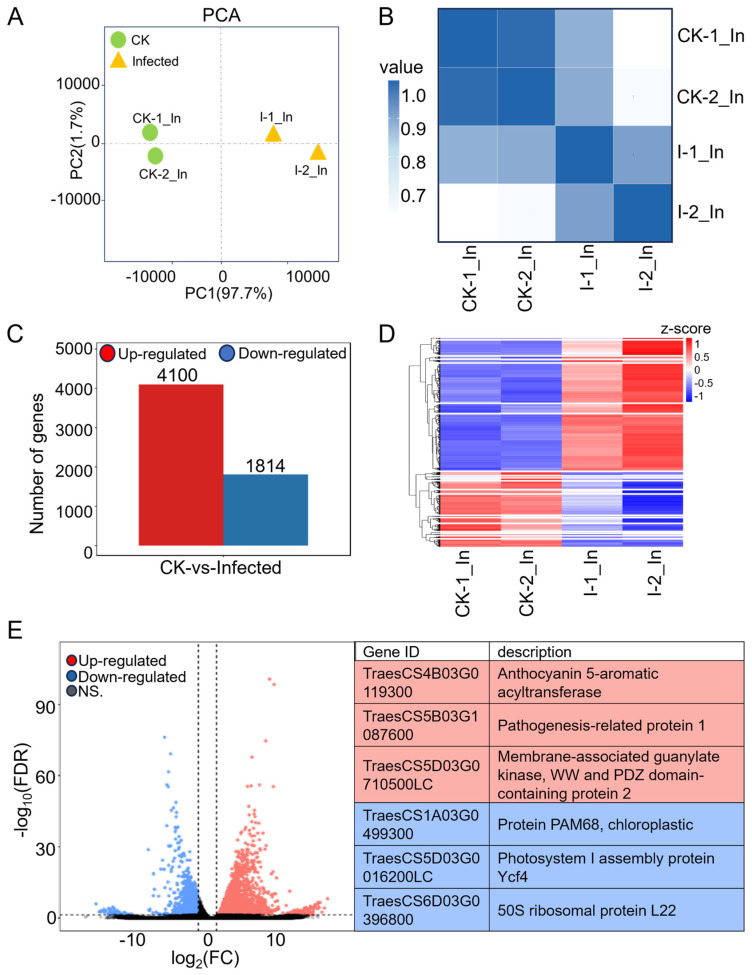
Comprehensive analysis of RNA-seq data from CK and infected wheat samples at 7 dpi. (**A**) Principal component analysis (PCA) of CK groups (green dots) and infected groups (yellow triangles) with two biological replicates each in RNA-seq. (**B**) Heatmap of Pearson correlation coefficients across four samples. The gradient of colors, transitioning from white to dark blue, represents the correlation strength between each pair of samples, with darker shades indicating stronger correlations. (**C**) Numbers of differentially expressed genes (DEGs) in wheat after *Pst* infection with |Log_2_ (FC)| > 1 and FDR (false discovery rate) < 0.05. (**D**) Heatmap of DEGs with 20 clusters. Rows, individual mRNA transcripts; columns, individual CK and infected samples. Red and blue represent up-regulation and down-regulation of mRNA levels in CK and infected samples, respectively. (**E**) Volcano plot showing up-regulated genes (red) and down-regulated genes (blue) in wheat with *Pst* infection. The gene ID and corresponding gene descriptions for the three most significantly up-regulated genes as well as the three most significantly down-regulated genes are provided.

RNA-seq data from two biological replicates each from CK and infected samples, after filtering for adapters (Table S1), quality control (Table S2), and removal of rRNA (Table S3), were aligned to the wheat reference genome (IWGSC CS RefSeq v2.1, https://www.ncbi.nlm.nih.gov/datasets/genome/GCF_018294505.1 (accessed on 10 September 2023)) (Table S4), producing approximately 37–61 million reads for each of the four samples. Principal component analysis (PCA) of the four samples, considering the distribution of sample points along PC1 and PC2, showed that I-1_In and I-2_In were closely related, as were CK-1_In and CK-2_In (Figure 2A). Pearson correlation analysis of the four samples, shown in a heatmap (Figure 2B), revealed that both the correlation coefficient between I-1_In and I-2_In and between CK-1_In and CK-2_In exceeded 0.9. Integrating the *PsEF1* expression data from the two biological replicates (Figure 1D), the sequencing quality control, and genome alignment results (Tables S1–S4), along with the PCA and Pearson correlation analysis (Figure 2A,B), we demonstrated the reproducibility of the data, which are suitable for further analysis.

### 2.2. Most Differentially Expressed Genes (DEGs) in Wheat Were Induced by Pst Infection, while a Significant Decrease Was Observed in Genes Related to Photosynthesis

Expression levels were first presented as a raw read count, which indicates the number of reads corresponding to a transcript. However, the raw read count is influenced by sequencing depth and gene length, making it unsuitable for comparing differential gene expression across samples. To ensure accuracy in subsequent analyses, we normalized the sequencing depth, gene, or transcript length to obtain the TPM (transcript per kilobase per million mapped reads) for each gene in four samples before further analysis (Table S5). Without considering the FDR (false discovery rate), comparing TPM values of infected and CK samples revealed a fold change (FC), with log_2_(FC) > 0 indicating 69,083 genes with up-regulation in the infected sample and log_2_(FC) < 0 indicating 28,424 genes with down-regulation. After further filtering for genes with |log_2_(FC)| > 1 and FDR < 0.05, the differentially expressed genes (Table S6) were identified, 4100 up-regulated and 1814 down-regulated genes (Figure 2C), showing that most differentially expressed genes in wheat were induced by the pathogen.

Hierarchical clustering was performed on the expression patterns of 5914 differentially expressed genes (Table S7), categorizing them into 20 clusters, and the clustering results are displayed in the heatmap (Figure 2D). These genes, with similar expression patterns, may have common functions or be involved in the same metabolic pathways and signaling pathways. Among them, cluster No. 20, which is enriched with the highest number of differentially expressed genes, involves 1010 genes that are in the pathogen-infected samples. Within these 1010 genes, 13 genes are annotated in the KEGG pathway as a “plant-pathogen interaction”, including Calmodulin (*TraesCS1B03G1255400*, *TraesCS5B03G0150400*, *TraesCS7B03G1109900*, *TraesCS7D03G1164400*), NBS-LRR disease resistance protein (*TraesCS1A03G0059400*, *TraesCS7D03G0011300*), pathogenesis-related protein 1 (*TraesCS5B03G1087600*, *TraesCS7D03G0450000*), 3-ketoacyl-CoA synthase (*TraesCS4A03G0012800*), calcium-dependent protein kinase (*TraesCS4A03G0728000*), cyclic nucleotide-gated channel (*TraesCS5D03G0900900*), protein kinase family protein (*TraesCS6B03G0271200*), and chaperone protein htpG family protein (*TraesCS7A03G1288100*), indicating genes related to calcium ion channel regulation in plant immunity, showing a degree of a coordinated response to combat *Pst* invasion.

The significantly expressed genes with up- and down-regulations in comparison to CK are presented in the volcano plot with FDR values. Many of the genes appear within the fold change values of 1 < |log_2_(FC)| < 5, having relatively low FDR values, with highly credible differential changes. The three most significantly up-regulated genes are *TraesCS4B03G0119300*, *TraesCS5B03G1087600*, and *TraesCS5D03G0710500LC*; the most significantly down-regulated genes are *TraesCS1A03G0499300*, *TraesCS5D03G0016200LC*, and *TraesCS6D03G0396800* (Figure 2E). Among the up-regulated genes, *TraesCS5B03G1087600*, a pathogenesis-related protein 1 (PR1), is considered an important defense protein. The production and accumulation of plant PR proteins are key responses to various biotic and abiotic stresses [37]. The two most significantly down-regulated genes are related to photosynthesis. In summary, wheat RNA expression was mostly up-regulated by *Pst*.

**Figure 3:**
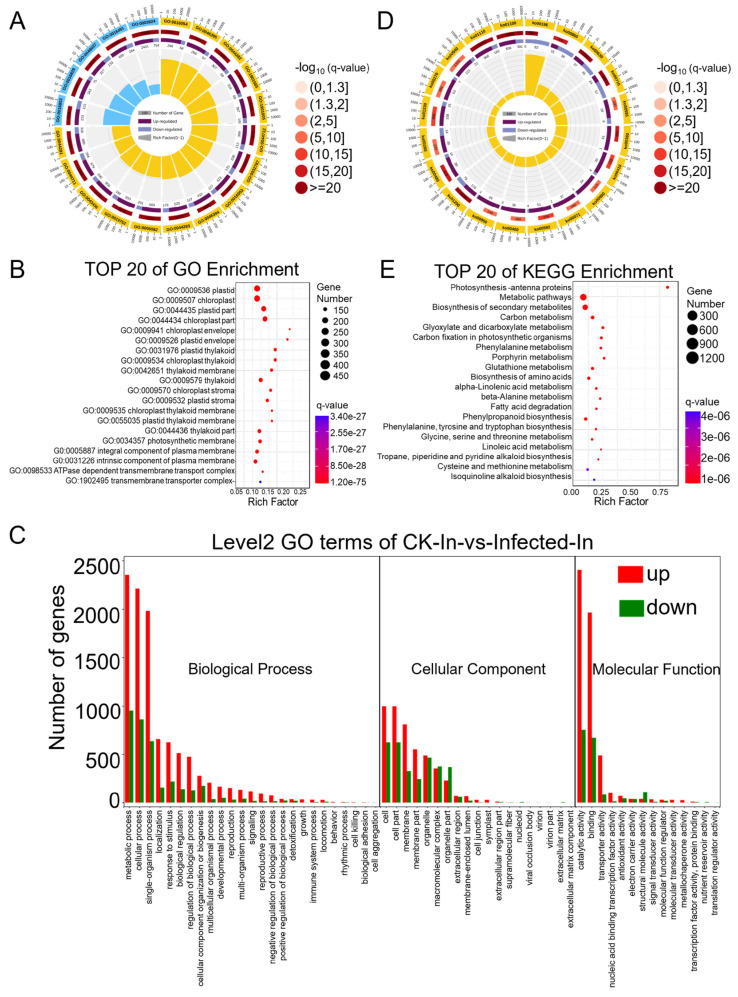
GO (Gene Ontology) and KEGG (Kyoto Encyclopedia of Genes and Genomes) pathway enrichment analysis of differentially expressed genes (DEGs) identified based on the RNA-seq data from CK and *Pst*-infected wheat leaves. (**A**) GO enrichment circle diagram of differentially expressed genes (DEGs). From outer circle to inner circle: Circle 1: The top 20 enriched GO terms of DEGs, and the coordinate scale outside the circle is the number of DEGs. Yellow and blue represent the GO terms of biological process and molecular function, respectively. Circle 2: The background of DEGs enriched in each GO term. The greater the number of DEGs, the longer the bar, and the smaller the *q*-value, the redder the color. Circle 3: The bar of the proportion of up-regulated (dark purple) and down-regulated (light purple) DEGs. The specific values are shown below. Circle 4: The rich factor value of each GO term (the number of DEGs divided by the total number of genes in the GO term); each grid line of the background grid represents 0.1. (**B**) Bubble chart illustrating the top 20 enriched GO terms in the cellular component category of DEGs based on *q*-value, with GO term ID and annotation on the y-axis and rich factor on the *x*-axis. (**C**) The histogram of level 2 GO terms enrichment classification of DEGs in infected samples in comparison to CK samples. The histogram depicts the number of up-regulated (in red) and down-regulated (in green) genes in each GO term. (**D**) KEGG pathway enrichment circle diagram of DEGs. From outer circle to inner circle: Circle 1: The top 20 enriched A class KEGG pathways of DEGs, and the coordinate scale outside the circle is the number of DEGs. Yellow represents the KEGG pathway of metabolism. Circle 2: The background of DEGs enriched in each pathway. The greater the number of DEGs, the longer the bar, and the smaller the *q*-value, the redder the color. Circle 3: The bar of the proportion of up-regulated (dark purple) and down-regulated (light purple) DEGs enriched in each pathway. The specific values are shown below. Circle 4: The rich factor value of each pathway (the number of DEGs divided by the total number of genes in the pathway); each grid line of the background grid represents 0.1. (**E**) Bubble chart illustrating the top 20 enriched KEGG pathways of DEGs based on *q*-value, with KEGG term annotation on the y-axis and rich factor on the x-axis.

Differential genes were mapped to terms in the Gene Ontology (GO) database (http://www.geneontology.org/ (accessed on 11 October 2022)) as a molecular function, cellular component, and biological process. This mapping allowed for the determination of the number of differentially expressed genes per term, yielding lists of differential genes associated with specific GO functions. A hypergeometric test was then applied to identify GO terms significantly enriched among the differentially expressed genes in comparison to the background, resulting in 3204 terms enriched in biological process, 1611 in molecular function, and 540 in cellular component (Tables S8–S10). The top twenty enriched GO terms are presented in a GO enrichment circle plot (Figure 3A), with fifteen items enriched in biological process and five in molecular function. Each GO term predominantly featured more up-regulated than down-regulated genes, consistent with the overall changes observed in differentially expressed genes. Specifically, the biological process GO terms GO:0016054 (organic acid catabolic process), GO:0046395 (carboxylic acid catabolic process), and GO:0044282 (small molecule catabolic process) and the molecular function GO terms GO:0016853 (isomerase activity) and GO:0016829 (lyase activity) showed a lower *q*-value and a higher rich factor, contributing to high significance. Due to fewer entries in the cellular component, this GO term was not noticeable in the enrichment circle plot. Therefore, the top 20 enriched items under cellular component were further highlighted (Figure 3B), showing clear enrichment in terms such as “plastid”, “chloroplast”, and “thylakoid”, indicating a relation to photosynthesis. To compare the ratio of up-regulated to down-regulated genes within the main GO terms of biological process, molecular function, and cellular component (Table S11), a GO enrichment bar chart was created (Figure 3C). Integrating RNA-Seq data analysis, 24 annotations for 35 genes were identified in the GO term related to the “immune system process”, elucidating the interplay of genes involved in plant immune/defense mechanisms against pathogens (Table 1. In most GO terms, the number of up-regulated differential genes exceeded that of down-regulated ones, except for a few terms where down-regulated genes were more numerous: GO:0032991 (macromolecular complex), GO:0044422 (organelle part), GO:0099512 (supramolecular fiber), GO:0009295 (nucleoid), GO:0031012 (extracellular matrix), GO:0044420 (extracellular matrix component), GO:0005198 (structural molecule activity), GO:0045735 (nutrient reservoir activity), and GO:0045182 (translation regulator activity).

Pathway analysis based on KEGG (Kyoto Encyclopedia of Genes and Genomes), a major public database for pathways, aids in further understanding the biological functions of genes. Pathway significance enrichment analysis uses KEGG pathways as units and applies a hypergeometric test to identify pathways significantly enriched among the differential genes in comparison to the entire background. This analysis can determine the primary biochemical metabolic and signal transduction pathways in which the genes are involved (Table S12), enriching a total of 121 KEGG A class pathways, with 97 annotated as metabolism. The list of the “plant-pathogen interaction pathway” through RNA-Seq data and KEGG revealed 18 annotations for 80 genes, highlighting the dynamic battle where increased pathogen virulence exerts selective pressure on plants to enhance or alter their immune/defense mechanisms (Table 2). The top 20 enriched KEGG pathways are presented in a KEGG enrichment circle plot (Figure 3D), and those with the smallest *q*-values are depicted (Figure 3E). Notably, the pathway KO00196 (photosynthesis-antenna proteins) had the highest rich factor, and all the enriched genes were significantly down-regulated. In summary, following infection by *Pst*, wheat’s transcriptome was predominantly up-regulated, with fewer pathways of down-regulated genes. In particular, genes related to photosynthesis were notably and comprehensively down-regulated.

### 2.3. The Landscape of m^6^A Methylome in Pst-Infected Wheat Leaves

Methylated RNA immunoprecipitation (MeRIP) is based on the principle of recognizing and binding m^6^A-modified RNA with m^6^A-specific antibodies, enriching methylated fragments through RNA immunoprecipitation, and combining this with high-throughput sequencing to study regions of RNA undergoing m^6^A methylation modification across the entire transcriptome (Figure 1A). Partial RNA from four samples for RNA-seq, CK-1, CK-2, I-1, and I-2, underwent enrichment and elution with m^6^A-specific antibodies for MeRIP-seq analysis. After filtering low-quality data and adapters (Table S13), quality control (Table S14), and removal of rRNA (Table S15), the data were aligned to the wheat reference genome (Table S16), resulting in approximately 41–54 million reads for each of the four samples. Peak analysis identified regions and sites of RNA with m^6^A modification across the whole genome. Using the R package exomePeak2 [38], peak scanning (peak calling) was conducted on a group basis across the genome with a default threshold of *p*-value < 0.05. This was followed by the analysis of the genomic location of peaks, peak region sequence information, and annotation of genes (Tables S17 and S18), leading to the screening and annotation of peak-associated genes (Tables S19 and S20).

**Figure 4:**
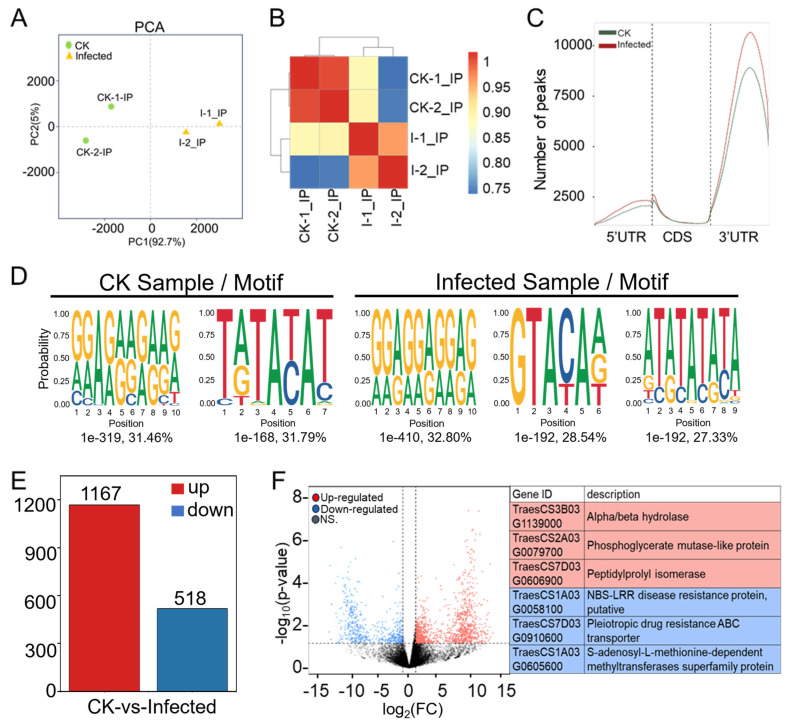
Comprehensive analysis of MeRIP-seq data from CK and infected wheat samples at 7 dpi. (**A**) Principal component analysis (PCA) of CK groups (green dots) and infected groups (yellow triangles) with two biological replicates each in MeRIP-seq. (**B**) Heatmap of Pearson correlation coefficients across four samples in MeRIP-seq. The color from blue to red represents the correlation from weak to strong. (**C**) Distribution and density of m^6^A peaks of CK sample and infected sample within 5′UTR (untranslated region), CDS (coding sequence), and 3′UTR. (**D**) Motif analysis of enriched RRACH and DRACH (R is A/G, H is A/U/C and D is A/G/U) conserved motifs for m^6^A peaks in wheat from CK and *Pst*-infected samples. (**E**) Numbers of differentially m^6^A-modified peaks (DMPs) in wheat after *Pst* infection with |log_2_(FC)| > 1 and *p*-value < 0.05. (**F**) Volcano plot showing up-regulated (red) and down-regulated (blue) m^6^A peak-related genes in wheat after *Pst* infection. The gene ID and corresponding gene descriptions for the three most significantly up-regulated peak-related genes as well as the three most significantly down-regulated peak-related genes are provided.

Principal component analysis (PCA) was performed using uniquely mapped reads, specifically by dividing the genome into 10 kb windows, counting the number of reads in each window, and reducing the read counts across all windows to a few principal components. PCA of the four MeRIP-seq samples, considering the distribution of sample points along PC1 and PC2, showed that I-1_In and I-2_In were closely related, as were CK-1_In and CK-2_In (Figure 4A). A heatmap was generated with the pairwise sample correlation using uniquely mapped reads by dividing the genome into 10 kb windows, counting the number of reads in each window, and then calculating the Pearson correlation coefficient for read counts across all windows. The Pearson correlation coefficients for both I-1_In vs. I-2_In and CK-1_In vs. CK-2_In were greater than 0.9. A higher correlation coefficient indicated a higher similarity in RNA methylation patterns between samples (Figure 4B). Integrating the *PsEF1* expression data from the two biological replicates (Figure 1D), the sequencing quality control, genome alignment, and peak calling results (Tables S13–S20), along with the PCA and Pearson correlation analysis (Figure 4A,B), demonstrated reproducibility among biological replicates, high sequencing quality, and alignment accuracy, making the MeRIP-seq sequencing data suitable for further analysis.

In CK and infected samples, the number of peaks was 15,249 and 17,864, respectively (Table S21); the infected ones not only showed a higher number of peaks in comparison to CK but also a greater chromosomal peak coverage (Figure S2). Further analysis of the distribution of peaks in the 5′ UTR (untranslated region), start codon, CDS (coding sequence), stop codon, and 3′ UTR (Figure 4C and Figure S3) showed that the number of peaks in infected samples was higher across different gene functional elements in comparison to CK, with the largest increases observed at the 5′ UTR and start codon, which were 40.34% and 30.37%, respectively. The smallest increase was in the 3′ UTR at 11.78%, but the changes in the 3′ UTR peaks were concentrated in the middle position of the 3′ UTR. Meanwhile, the peak distribution in this study shows a similar m^6^A modification distribution to other plant species, regardless of whether they are monocots or dicots, whether in fruits or other tissues or under some stress conditions [39].

The m^6^A modification is the most prevalent internal modification in eukaryotic mRNA, with m^6^A methylation being highly conserved, typically embedded in conserved consensus sequences like 5’-DRACH-3’ and 5’-RRACH-3’ (D = G/A/U, R = G/A, H = A/U/C). The frequency distribution of specific motifs (RRACH, DRACH) within all peaks across all samples was analyzed using HOMER to construct average base frequency matrices. In CK sample two, in the infected sample, three significant motifs were identified, two of which were very similar to the two motifs found in the control sample. A particular motif emerged as a unique methylation site motif, GTACAA, which might specifically be determined by *Pst* (Figure 4D). We found that 6174 genes with this motif were methylated in our experiments. These genes associated with disease resistance include RPP13-like, RGA2-like EDR2L, putative RGA1, and RPM1-like. Similarly, genes related to the immune response, including those involved in the immune system and effector processes, such as WD repeat-containing protein 7, RRP44A, MACPF domain-containing protein, and numerous others like Histone acetyltransferase and IAN9 (immune-associated nucleotide-binding protein 9), are essential for the robust functioning of the immune system. These genes were identified in a sample infected with *Pst* having the GTACAA motif, which is a novel methylation site linked to this pathogen. The discovery of this unique methylation motif indicates a specific epitranscriptional response to *Pst* infection, suggesting a sophisticated mechanism of plant–pathogen interaction and defense regulation (Figure 4D, Table S22).

### 2.4. The Majority of Wheat Genes Associated with Differentially Methylated Peaks (DMPs) Showed Hyper-Methylation in Response to Pst

RNA-seq data from “CK” and “Infected” samples (Table S5) and peak data (Tables S19 and S20) were merged, resulting in 22,629 merged peaks and their peak-related gene expression counts and annotations across the four sequencing samples (Table S23). TPM and *p*-values were further calculated. The filtering criteria of |log_2_(FC)| > 1 and *p*-value < 0.05 identified differentially expressed genes associated with differentially methylated peaks (DMP-related genes) (Table S24), totaling 1804 DMP-related genes. Of these, 1167 were up-regulated DMP-related genes (indicating hyper-methylation) and 518 were down-regulated DMP-related genes (indicating hypo-methylation) (Figure 4E), suggesting that most DMP-related genes exhibit a trend towards hyper-methylation upon *Pst* infection.

Volcano plot analysis was conducted for DMP-related genes comparing infected samples to CK samples (Figure 4F). The volcano plot displays the situation of differentially expressed DMP-related genes, with genes closer to edges showing greater differences. DMP-related genes around |log_2_(FC)| = 10 had relatively lower FDR values, indicating more credible and significant changes. The three most significantly hyper-regulated genes were *TraesCS3B03G1139000*, *TraesCS2A03G0079700*, and *TraesCS7D03G0606900*; the most significantly hypo-regulated genes were *TraesCS1A03G0058100*, *TraesCS7D03G0910600*, and *TraesCS1A03G0605600* (Figure 4F). Among the significantly hypo-regulated genes, *TraesCS1A03G0058100* is annotated as a putative NBS-LRR disease resistance protein. To our knowledge, this may be the first report of an NBS-LRR gene showing hypo-methylation after *Pst*–wheat compatible interaction, suggesting that the m^6^A mechanism could directly regulate key genes, such as resistance genes, in plant immunity.

**Figure 5:**
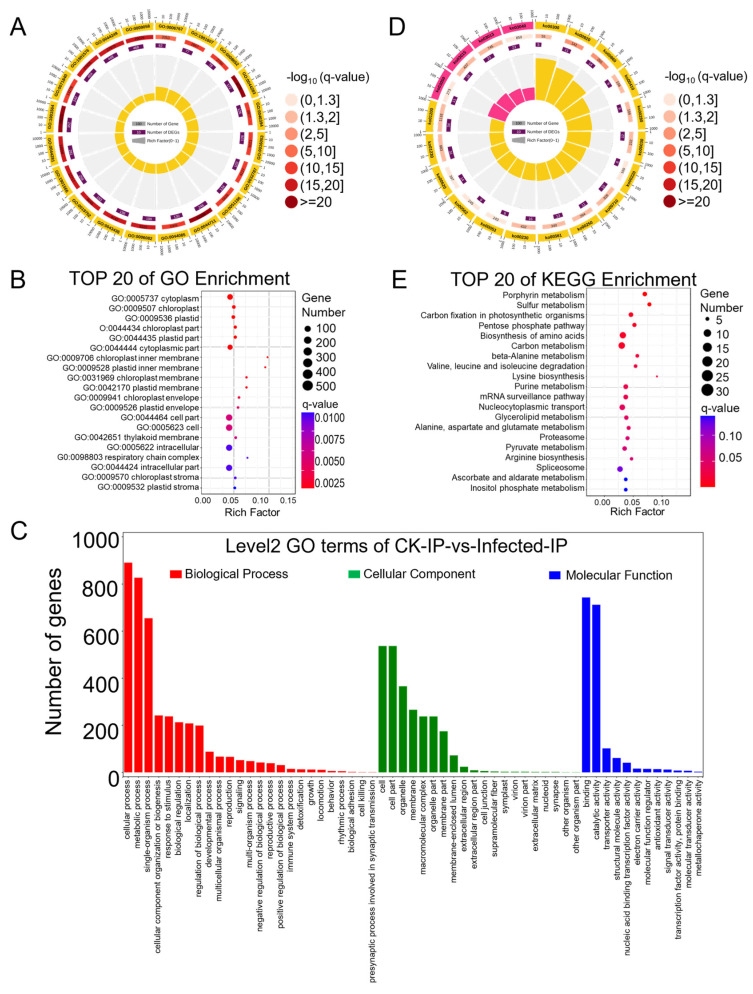
GO (Gene Ontology) and KEGG (Kyoto Encyclopedia of Genes and Genomes) pathway enrichment analysis of differentially m^6^A-modified peak (DMP)-related genes identified based on the MeRIP-seq data from *Pst*-infected wheat leaves in comparison to CK samples. (**A**) GO enrichment circle diagram of differentially m^6^A-modified peak (DMP)-related genes. From outer circle to inner circle: Circle 1: The top 20 enriched GO terms of DMP-related genes, and the coordinate scale outside the circle is the number of DMP-related genes. Yellow represents the GO term of biological process. Circle 2: The background of DMP-related genes enriched in each GO term. The greater the number of DMP-related genes, the longer the bar, and the smaller the *q*-value, the redder the color. Circle 3: The number of DMP-related genes enriched in each GO term. Circle 4: The rich factor value of each GO term (the number of DMP-related genes divided by the total number of genes in the GO term); each grid line of the background grid represents 0.1. (**B**) Bubble chart illustrating the top 20 enriched GO terms in the cellular component category of DMP-related genes based on *q*-value, with GO term ID and annotation on the y-axis and rich factor on the x-axis. (**C**) The histogram of level 2 GO terms enrichment classification of DMP-related genes in infected samples in comparison to CK samples. (**D**) KEGG pathway enrichment circle diagram of DMP-related genes. From outer circle to inner circle: Circle 1: The top 20 enriched A class KEGG pathways of DMP-related genes, and the coordinate scale outside the circle is the number of DMP-related genes. Yellow and pink represent the KEGG pathways of metabolism and genetic information processing, respectively. Circle 2: The background of DMP-related genes enriched in each pathway. The greater the number of DMP-related genes, the longer the bar, and the smaller the *q*-value, the redder the color. Circle 3: The number of DMP-related genes enriched in each pathway. Circle 4: The rich factor value of each pathway (the number of DMP-related genes divided by the total number of genes in the pathway); each grid line of the background grid represents 0.1. (**E**) Bubble chart illustrating the top 20 enriched KEGG pathways of DMP-related genes based on *q*-value, with KEGG term annotation on the y-axis and rich factor on the x-axis.

DMP-related genes were enriched in the GO categories of biological process, cellular component, and molecular function, with respective counts of 2838, 571, and 1274 (Tables S25–S27). The top 20 enriched GO terms, all from the biological process category (Table S25), were presented in a GO enrichment circle plot (Figure 5A), with a relatively high rich factor for GO:0006767 (water-soluble vitamin metabolic process), GO:1901607 (alpha-amino acid biosynthetic process), and GO:0008652 (cellular amino acid biosynthetic process) and a relatively low *q*-value for GO:1901564 (organonitrogen compound metabolic process), GO:0044711 (single-organism biosynthetic process), and GO:0044283 (small molecule biosynthetic process). The top 20 entries under the cellular component category (Table S26) showed enrichment as in the RNA-seq cellular component results, with DMP-related genes enriched in GO cellular component annotations related to photosynthesis, such as “chloroplast”, “plastid”, “thylakoid”, and “stroma” (Figure 5B). Further analysis of the quantity and annotations of DMP-related genes in the main GO terms of biological process, cellular component, and molecular function (Table S28) led to the creation of a GO enrichment bar chart (Figure 5C).

Significant enrichment in KEGG pathways identifies the primary biochemical metabolic and signal transduction pathways involved by DMP-related genes (Table S29), enriching a total of 115 KEGG A class pathways, with 84 annotated as metabolism. The top 20 enriched KEGG pathways are presented in the KEGG enrichment circle plot (Figure 5D); those with the smallest *q*-values are depicted in a bubble chart (Figure 5E). In the KEGG enrichment circle plot, sixteen entries were enriched in metabolism and four in genetic information processing. The bubble chart shows that aside from the first 14 pathways with *q*-values less than 0.05 indicating significance, the *q*-values of other enriched pathways were greater than 0.05, suggesting lower significance. The pathways with higher enrichment factors and smaller *q*-values include KO00860 (porphyrin metabolism), KO00920 (sulfur metabolism), KO00030 (pentose phosphate pathway), KO00410 (beta-alanine metabolism), KO00280 (valine, leucine, and isoleucine degradation), and KO00300 (lysine biosynthesis). This suggests that in wheat, upon compatible interaction with stripe rust, these pathways of secondary metabolism were regulated by the m^6^A mechanism, thereby affecting wheat’s growth and development.

### 2.5. Association Analysis of DEGs and DMP-Related Genes in Pst-Infected Leaves in Comparison to CK

**Figure 6:**
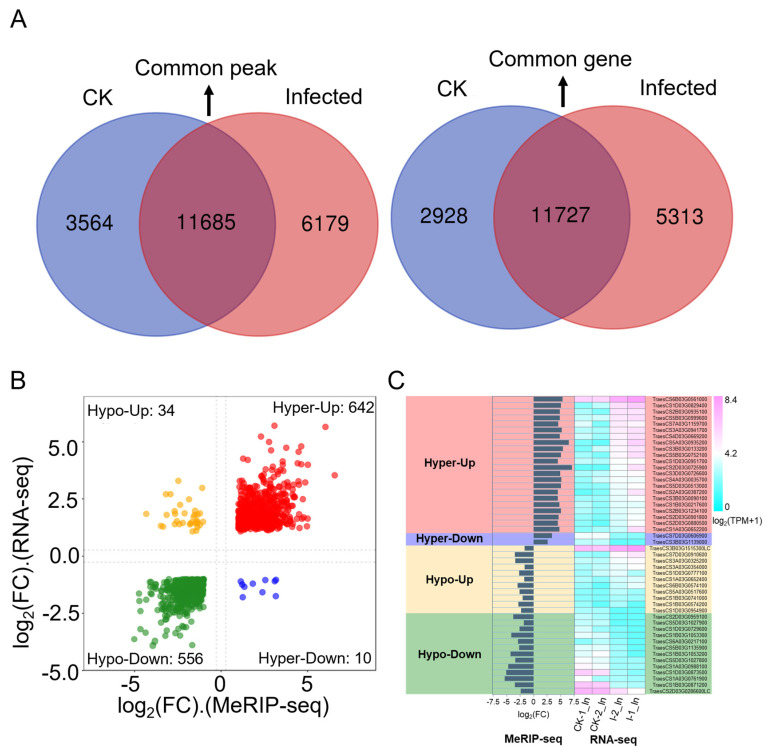
Conjoint analysis of MeRIP-seq and RNA-seq data. (**A**) Dual Venn diagrams of m^6^A-modified peaks and their corresponding genes in CK and infected samples. Left: numbers of unique peaks in CK and infected samples, along with common peaks. Right: numbers of these peaks represent genes of the two groups. (**B**) A four-quadrant diagram illustrating significant changes in DEGs and DMP-related genes, comparing infected samples to CK. |log_2_(FC)| > 1 in both MeRIP-seq and RNA-seq; FC is fold change. (**C**) Bar chart and heat map of the hyper-up, hyper-down, hypo-up, and hypo-down genes are shown in Table 3.

To further clarify the relationship between gene expression levels and m^6^A modifications, an analysis was conducted based on the number of genes (Table S5) and the number of peaks (Table S23) detected in both CK and infected samples from combined RNA-seq and MeRIP-seq data, leading to Figure 6A. Comparing the number of m^6^A modification peaks between CK and infected samples, there were 11,685 common peaks, 3565 unique peaks in CK samples, and 6179 unique peaks in infected samples, showing an approximate 73% increase in peak types, indicating that m^6^A modifications increase due to infection (Figure 6A, left panel). Further analysis of the number of genes associated with these peaks revealed 11,727 common genes, 2928 unique genes in CK samples, and 5313 unique genes in infected samples (Figure 6A, right panel). Typically, one or more peaks map to a gene, so the number of genes associated with peaks is less than the number of peaks. In this study, the total number of peaks in CK and infected samples was 21,428, with 19,968 genes associated with peaks, aligning with the expected mapping ratio between peaks and genes (Figure 6A). However, the number of common genes is slightly higher than the number of common peaks, possibly because most peaks are not regulated upon *Pst* infection associated with genes having only one m^6^A modification peak, leading to a similar count of common genes and peaks. Also, a gene may have multiple transcripts, and if a peak maps to an overlapping part of a transcript, it could be associated with multiple transcripts, thus increasing the count of common genes over common peaks. Interestingly, the number of genes associated with peaks in both CK and infected samples is significantly lower than the number of peaks, suggesting that genes associated with unique peaks are more likely to be regulated by more than one m^6^A modification. Genes modified by multiple peaks are more likely to be involved in response to the *Pst* pathogen, and their numbers are substantial, indicating that the transcriptome is extensively regulated by the m^6^A mechanism by the pathogen.

To further correlate m^6^A modification with gene expression, a co-differential gene analysis was conducted. This involved combining differentially expressed genes from RNA-seq (Table S6) with differential m^6^A peaks from MeRIP-seq (Table S24) together (Table S30). By using a filter criterion that required |log_2_(FC)| > 1 in both RNA-seq and MeRIP-seq, an FDR < 0.05 in RNA-seq data, and a *p*-value < 0.05 in MeRIP-seq data, a total of 1242 significant co-differential genes were filtered (Table S31). A quadrant volcano plot allows for the presentation of the expression level and the methylation level changes together (Figure 6B). The largest group consisted of 642 genes with significant hyper-methylation and up-regulated gene expressions. Following this, 556 genes showed significant hypo-methylation with down-regulated expression. Additionally, 34 genes had significant hypo-methylation with up-regulated expression, and 10 genes had significant hyper-methylation with down-regulated expression.

To focus deeper on the annotation information and function of markedly co-differentiated genes, the filtering criteria were further tightened by adjusting |log_2_(FC)| > 1.5 in both RNA-seq and MeRIP-seq data, FDR < 0.05 in RNA-seq data, and a *p*-value < 0.05 in MeRIP-seq data. Under these new settings, 346 genes were identified in the hyper-up category, 212 in hypo-down, 10 in hypo-up, and 2 in hyper-down. For genes in the hyper-up and hypo-down categories, the ones with the lowest FDR and *p*-value were further selected. A total of 48 genes are presented in Table 3, along with gene IDs, annotations, expression patterns, peak distributions, peak start positions, IP_log_2_(FC) values, and Input_log_2_(FC) values. The expression levels of these 48 genes and the |log_2_(FC)| from MeRIP-seq are displayed as histograms, and |log_2_(TPM + 1)| from RNA-seq are shown as heatmaps in Figure 6C. Unlike the genes in the hyper-up category, the overall expression level of significantly up-regulated genes in the hypo-up category was lower across the transcriptome. In the hypo-down category, except for *TraesCS1D03G0954900* and *TraesCS2D03G0286600LC*, the other 12 significant co-differential genes were annotated as chlorophyll *a*/*b* binding proteins. Overall, this study found that by stripe rust invasion, most genes in wheat responded in a hyper-up manner, consistent with the observation that most genes in RNA-seq were up-regulated and most genes in MeRIP-seq showed hyper-methylation. A notable discovery was that photosynthesis-related genes, possibly due to the secretion of effectors or other pathogenic factors from *Pst*, might be involved in the regulation of the wheat m^6^A mechanism, leading to hypo-methylation modifications, decreased expression of photosynthesis-related genes, and impaired photosynthesis function, thus affecting basic biomolecular metabolism and secondary metabolite production, further reducing the wheat resistance to stripe rust and resulting in successful invasion.

### 2.6. Divergent Expression Dynamics of m^6^A Regulators in Response to Pst Invasion

**Figure 7:**
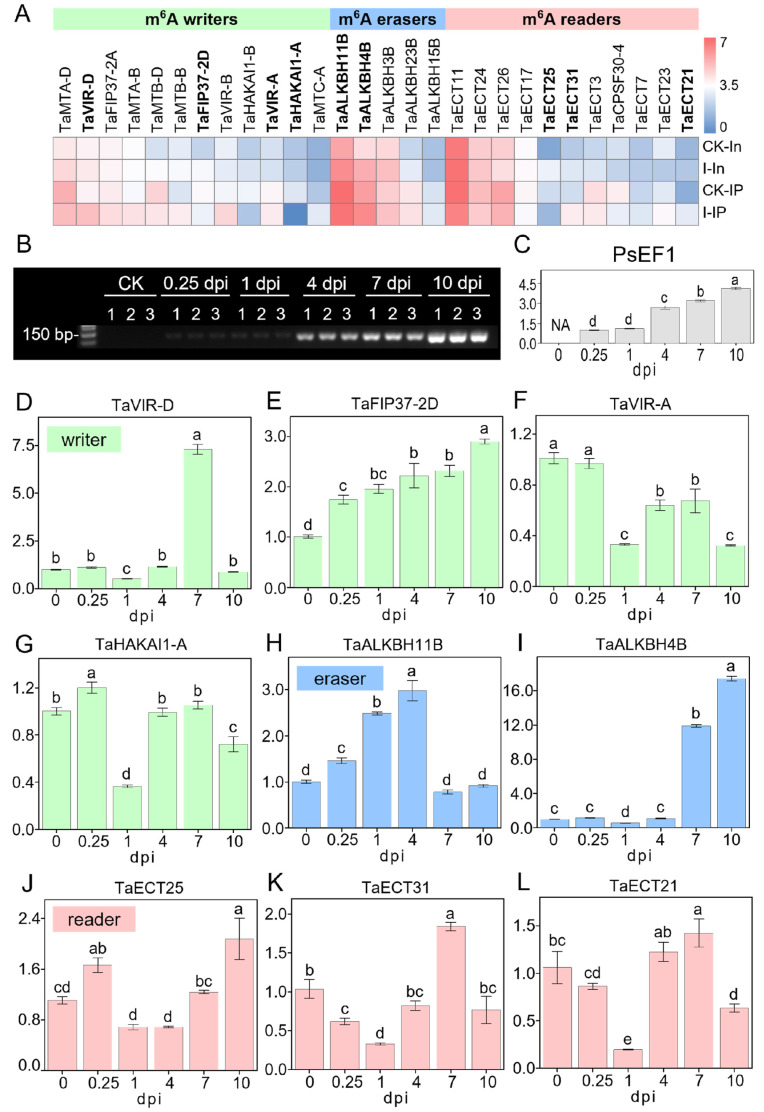
Expression patterns of m^6^A writers, erasers, and readers in wheat after *Pst* inoculation. (**A**) Heatmap illustrating the expression profiles of m^6^A writers, erasers, and readers from MeRIP-seq and RNA-seq data in CK and infected samples. The color scale within the dendrogram represents the log_2_(TPM + 1) of each m^6^A regulator gene. Gene names in bold were further analyzed in (**D**–**L**). (**B**) Agarose gel (2.5%) electrophoresis displaying the sqRT-PCR products of *PsEF1* in *Pst*-infected wheat leaves collected from 0 (CK), 0.25, 1, 4, 7, 10 dpi with three biological replicates. (**C**) Quantitative analysis of band intensity for sqRT-PCR products of *PsEF1*, as depicted in (**B**). Intensity values of all samples are normalized against the intensity value of the 0.25 dpi sample. (**D**–**L**) Relative expression levels of m^6^A regulators in *Pst*-infected wheat determined by RT-PCR. Each panel corresponds to a different m^6^A regulator gene: (**D**) *TaVIR-D*, (**E**) *TaFIP37-2D*, (**F**) *TaVIR-A*, (**G**) *TaHAKAI1-A*, (**H**) *TaALKBH11B*, (**I**) *TaALKBH4B*, (**J**) *TaECT25*, (**K**) *TaECT31*, and (**L**) *TaECT21*. Expression levels are normalized to those detected in the CK (0 dpi). The small case letters on the bars of the figures refer to the relative expression levels.

Since previous research rarely focused on the expression changes in m^6^A regulators in the wheat–*Pst* pathosystem, we further analyzed the expression patterns of m^6^A regulators in RNA-seq and MeRIP-seq data. According to a review by Yue et al., (2019) [40], which analyzed and annotated 90 wheat m^6^A regulator genes, including 20 m^6^A writers, 29 m^6^A erasers, and 41 m^6^A readers (Table S32), 28 of these m^6^A regulator genes were detectable in our RNA-seq and MeRIP-seq data, consisting of 12 m^6^A writers, 5 m^6^A erasers, and 11 m^6^A readers (Figure 7A). We further combined the average of TPM + 1 from two biological replicates of CK and infected samples in RNA-seq and MeRIP-seq and created a heatmap after converting to log_2_(TPM + 1) (Figure 7A). The heatmap shows that these 28 m^6^A regulator genes exhibit a variety of different expression patterns, whether they are m^6^A writers, m^6^A erasers, or m^6^A readers, or in terms of their expression patterns in RNA-seq and MeRIP-seq, indicating that the m^6^A regulatory mechanism in wheat–*Pst* interactions is complex and diverse. However, it is still possible to observe that some m^6^A regulator genes have relatively similar expression patterns in both RNA-seq and MeRIP-seq data, as well as in terms of expression levels, such as *TaHAKAI1-A* and *TaHAKAI1-B* among m^6^A writers, *TaALKBH3B* and *TaALKBH4B* among m^6^A erasers, and *TaECT21* and *TaECT31* among m^6^A readers. To further understand the temporal changes of m^6^A regulators after virulent *Pst* infection, we selected four writers, two erasers, and three readers for time-dependent expression analysis (Figure 1A). To test the changes in expression levels of m^6^A regulators after *Pst* infection, wheat materials without infection were collected as CK (also noted as 0 dpi) and at 0.25, 1, 4, 7, and 10 dpi after infection, with three biological replicates each. sqRT-PCR (semi-quantitative reverse transcription and polymerase chain reaction) products of the *PsEF1* gene in each sample showed that the infection was successful, with the intensity of the bands increasing over time and consistent across the three biological replicates (Figure 7B). To determine the relative expression levels of the *PsEF1* gene, the intensity of the bands in Figure 7B was analyzed using ImageJ (Figure 7C and Figure S1B). In the early stages of infection (0.25, 1 dpi), the expression levels of *PsEF1* were lower, indicating no significant proliferation of *Pst*, but to some extent, transcriptomic activity was already present due to the formation of haustoria. As the infection progressed, the amplification of *PsEF1* significantly increased, reflecting the clear increase in the biomass of stripe rust with the duration of infection, indicating the successful colonization in the host by overcoming its immune system.

Figure 7D–L show the relative expression levels of nine m^6^A regulators over time as the infection progresses using the qRT-PCR (Real-Time Quantitative Reverse Transcription PCR) assay, revealing that different m^6^A regulators exhibit various dynamic response patterns during the wheat response to *Pst* infection. For the two eraser genes, *TaALKBH11B* and *TaALKBH4B* (Figure 7H,I), it was observed that *TaALKBH11B* showed a clear response and induced expression in the early and initial stages of infection (0.25, 1, 4 dpi), while *TaALKBH4B* was clearly responsive and induced expression during the colonization phase (7, 10 dpi) of *Pst* inoculation. The three m^6^A reader genes detected all showed lower relative expression levels at 1 dpi (Figure 7J–L), which may be due to the lower response of m^6^A writer genes at 1 dpi (Figure 7D,F,G), indicating that the expression of m^6^A readers is subject to feedback regulation by the function of m^6^A writers. Unlike other writer genes, *TaFIP37-2D* continuously increased its induced expression throughout the infection stages (Figure 7E).

## 3. Discussion

This study combines RNA-seq and MeRIP-seq to analyze genes with significant differences in mRNA and m^6^A modifications in wheat after compatible *Pst* interaction transcriptome-wide. We also examined the temporal changes in wheat m^6^A regulators after *Pst* infection through qRT-PCR analysis (Figure 1A). From the perspective of m^6^A regulation, this study elucidates how wheat responds to *Pst* infection and how stripe rust interferes with wheat growth, metabolism, and immune pathways to successfully colonize. This research provides an important theoretical basis for understanding the disruption of m^6^A regulatory mechanisms and the genes regulated by the m^6^A mechanism during the infection process of biotrophic fungi in plants.

Regarding the omics studies on the interaction between *Pst* and wheat, with the development of sequencing technologies, improved genome assembly and annotation for wheat [41] and *Pst* (https://www.ncbi.nlm.nih.gov/datasets/genome/?taxon=27350 (accessed on 15 February 2024)) have led to the accumulation of data. Our research previously reviewed some of the transcriptome, microarray, and proteome omics analyses of wheat and *Pst*, analyzing potential *Pst* effector candidates [42,43]. Recently, the study of the pan-genome of *Pst* has laid the foundation for the population genetics and comparative genomics research of the *Pst* population [44]. Currently, dual RNA sequencing seems more suitable for samples that cannot be separated from the host wheat after *Pst* infection. This sequencing technology uses unique molecular identifier (UMI) tags for sequencing, which involves several more rounds of amplification of the reverse-transcribed templates than other sequencing methods. This approach optimizes the issue where plant RNA is abundant and pathogen RNA is relatively less abundant and harder to detect in plant samples after pathogen infection [45]. It is known that RNA has over 100 modifications, with m^6^A methylation being the most common modification in eukaryotic mRNA. Studies have found that m^6^A modification is involved in plant responses to biotic or abiotic stress, but there have been no reports on wheat response to *Pst* infection in this aspect. Therefore, this study combines RNA-seq and MeRIP-seq data to analyze the RNA response and m^6^A modification in wheat after virulent infection with stripe rust.

Research on photosynthesis and its related genes in the interaction between stripe rust and wheat has been reported from various perspectives. Chen et al. (2015) [46] found that after infection by stripe rust, resistant wheat had higher levels of D1 protein and light-harvesting complex II (LHCII) accumulation compared to susceptible wheat. The light-harvesting antenna protein CP29 in both resistant and susceptible wheat was phosphorylated under stripe rust infection, with more significant phosphorylation in resistant wheat. The thylakoid membranes in susceptible wheat suffered more extensive damage [46]. In this study, we also found a significant decrease in the expression of many light-harvesting antenna protein genes. Wang et al. (2019) discovered that the peripheral protein of photosystem II, PsbO, could be regulated by phosphorylation by a protein kinase WKS1 encoded by YR36, inhibiting ascorbate peroxidase, leading to the accumulation of substrate hydrogen peroxide, initially inhibiting the growth of the pathogen. Subsequently, photosystem II, without the protection of PsbO, acts as a source of hydrogen peroxide production in the broad-spectrum disease resistance process, inhibiting the reproduction of stripe rust and demonstrating plant resistance [47]. During the normal photosynthesis process, zeaxanthin epoxidase 1 (ZEP1) can transfer singlet oxygen to zeaxanthin, producing violaxanthin, avoiding the accumulation of reactive oxygen species and protecting the photosynthetic system. Chang et al. (2023) found that *ZEP1* mutation could inhibit the growth of stripe rust, promote the accumulation of reactive oxygen species at the invasion site, and significantly enhance wheat resistance [48]. Xu et al. (2019) discovered that the effector protein Pst_12806 could be transported to chloroplasts, where it interacts with the Rieske structure of the plant cytochrome *b*_6_*f* protein subunit *TaISP* (iron–sulfur protein), weakening the electron transfer capability of the *TaISP* Rieske domain during photosynthesis, thereby disrupting plant photosynthesis, inhibiting chloroplast-mediated reactive oxygen species, and further promoting pathogen colonization in the plant [49]. In this study, we found three genes of Rieske-type iron–sulfur protein enriched in the level 2 GO term annotated as an immune system process, and their m^6^A modifications showed significant changes after stripe rust infection. Andac et al. (2020) also identified a stripe rust effector, PstCTE1, which, despite lacking a clear conserved transport signal peptide as predicted by TargetP and ChloroP, was found to localize in chloroplasts regardless of whether the N-terminus was tagged [50]. The discovery of *Pst* effectors Pst_12806 and PstCTE1 suggests that disrupting host wheat photosynthetic machinery proteins is one of the strategies for *Pst* to infect the host. The above research on photosynthesis-related mechanisms in the interaction between stripe rust and wheat mostly focused on post-translational modifications. In contrast, this study provides new insights into the level of post-transcriptional regulation, specifically m^6^A modification of RNA. We found that photosynthesis-related genes with m^6^A modifications mostly showed hypo-methylation after stripe rust infection (Table S24), particularly among the most significantly hypo-methylated genes, most of which are related to photosynthesis (Figure 5B, Table 1 and Table S28). Thus, on one hand, the expression of photosynthesis-related genes may decrease, leading to a reduction in related proteins, and on the other hand, the regulation of m^6^A modifications may also interfere with the translation level of related proteins, ultimately leading to reduced photosynthesis, decreased substance accumulation, and increased plant susceptibility. Further research on photosynthesis and plant immunity is needed at the molecular level. Photosynthesis is a complex and precise regulatory mechanism, and genetic modification of a few photosynthesis-related genes might enhance disease resistance. However, the balance of the photosynthesis mechanism itself is easily disturbed and broken. Since photosynthesis is closely related to yield and specific agricultural practices do not wish to sacrifice yield for increased disease resistance, seeking a balance between the two is necessary.

## 4. Materials and Methods

### 4.1. Plant Materials and Pst Inoculation

The wheat cultivar MingXian169 (MX169) was cultivated in a growth chamber with a light intensity of 200 μmol·m^−2^·s, temperature maintained at 18 °C, and humidity at 80%. The light–dark cycle was set at 16 h of light and 8 h of darkness. When MX169 reached the two-leaf stage with fully expanded leaves, it was used for inoculation with *Pst* spores. Spores of Chinese yellow rust race CYR32 stored at −80 °C were used, and they were incubated in a 42 °C water bath for 10 min. Wheat leaves were sprayed with 2 mg/mL of Novec 7100, followed by the inoculation of spores. Infected MX169 were kept in darkness at 12 °C with 100% humidity for 1 d, then transferred to a growth chamber under normal conditions. After 2 weeks, the leaves showed abundant fresh spores, which were used to inoculate wheat cultivar Avocet/Yr7 (Avocet/Yr7 the stripe rust Avocet differential lines were originally generated by C.R. Wellings of Plant Breeding Institute, Narrabri, Australia). Inoculation conditions were the same as those for MX169. Infected Avocet/Yr7 were collected at 0.25, 1, 4, 7, and 10 dpi. Avocet/Yr7 only sprayed with 2 mg/mL of Novec 7100 solution without inoculation were used as control (CK) samples. Three biological replicates were collected for both CK and infected samples with different time points and then stored at −80 °C for subsequent RNA extraction.

### 4.2. RNA Extraction and Library Construction for RNA-Seq and MeRIP-Seq

Total RNA was extracted from samples stored at −80 °C using the TRIzol reagent (Life Technologies, Carlsbad, CA, USA, cat15596026) with the user guide of the manufacturer involving the homogenization of leaf tissue, phase separation with chloroform, RNA precipitation, and purification. Extracted RNA quality was assessed using the Agilent 2100 Bioanalyzer (Agilent Technologies, Santa Clara, CA, USA), ensuring suitability for library preparation and sequencing. Then, mRNA enriched by Oligo(dT) magnetic beads was fragmented into short fragments using a fragmentation buffer. Fragmented RNA was divided into two parts, one of which was used as the input (no immunoprecipitation experiment was performed). The other RNA enriched with an m^6^A-specific antibody was used as the IP. The input RNA and IP RNA were reversely transcribed into cDNA with random primers using NEBNext^®^ Ultra^TM^ RNA Library Prep Kit for Illumina (New England Biolabs, MA, USA catE7530). Next, the cDNA fragments were end-repaired, “A” base-tailed, and ligated to Illumina sequencing universal adapters. Finally, the qualified cDNA library with PCR amplification was sequenced using Illumina Novaseq6000 by GENE DENOVO Biotechnology Co., Ltd. (Guangzhou, China).

### 4.3. Data Analysis of Sequencing

In preprocessing RNA-seq and MeRIP-seq raw data, FASTQ v0.18.0 [51] was employed as a trimming adapter, filtering reads exceeding 10% N content, removing poly A sequences, and discarding low-quality reads. High-quality clean reads were aligned to the species ribosome database using Bowtie2 [52], with ribosomal RNA reads subsequently removed, and HISAT2 [53] was used for genome alignment. Differential gene expression was identified using criteria of FDR < 0.05 and |log_2_(FC)| > 1, classifying genes with significant changes in expression. PCA was performed with the R package models (http://www.r-project.org/ (accessed on 8 October 2022)). Differential RNA methylation was analyzed with DiffBind v1.20.0 [54], identifying peaks as significantly differential with *p*-value < 0.05 and |log_2_(FC)| > 1. StringTie v1.3.1 was utilized to assemble RNA-seq data from each sample against a known genome [55,56]. RSEM calculated TPM (transcript per kilobase per million mapped reads) values to quantify RNA transcript expression levels, enabling accurate across-sample comparisons [57]. GO [58] (http://www.geneontology.org/ (accessed on 11 October 2022)) and KEGG [59] enrichment was analyzed to clarify the function of differentially expressed genes. Using MACS2 [60] (version 2.1.2), read-enriched regions in MeRIP-seq data were identified through a dynamic Poisson distribution, defining peaks with a *q*-value < 0.05 and retaining those with over 50% overlap in at least two of the biological duplicates for further analysis. By analyzing the genomic location and gene annotation of peaks, related genes were confirmed, and the distribution of peaks across functional regions like 5′ UTR, CDS, and 3′ UTR was assessed [61]. Motif analysis was performed by HOMER [62], and significant motif identification in peak-associated transcripts was carried out by the MEME Suite (http://meme-suite.org/meme/ (accessed on 6 October 2022)) and DREME (http://meme-suite.org/tools/dreme (accessed on 6 October 2022)). The clustering heatmap (http://cloud.oebiotech.com/task/detail/heatmap/?id=31 (accessed on 11 February 2024)) and quadrant image (www.omicstudio.cn/tool/31 (accessed on 21 January 2024)) were formed using online software.

### 4.4. sqRT-PCR and qRT-PCR

After adjusting the concentration of RNA extracted from CK and 0.25, 1, 4, 7, and 10 dpi infected samples were ensured to be the same as 350 ng/μL. The genomic DNA was removed and synthesis of the first cDNA strand was performed according to the specifications of SweScript RT II First Strand cDNA Synthesis Kit (Servicebio, Wuhan China catG3333). A 10 μL genome removal reaction mixture was prepared with 8 μL RNA adjusted to 350 ng/μL, 1 μL 10×gDNA remover buffer, and 1 μL gDNA remover, lightly mixed and briefly centrifuged, then incubated at 37 °C for 2 min and placed on ice for later use. Then, 20 μL reverse transcription reaction mixture was prepared, including 10 μL genome removal mixture, 3 μL nuclease-free water, 4 μL 5 × reaction buffer, 1 μL 100 μmol/L oligo (dT)_18_ primer, 1 μL 100 μmol/L random hexamer primer, and 1 μL SweScript RT II enzyme mix, lightly mixed and briefly centrifuged, then incubated at 25 °C for 5 min, 55 °C for 15 min, and 85 °C for 5 s. The synthesized cDNA was stored at −20 °C.

A 20 μL sqRT-PCR mixture was prepared, which included 1 μL cDNA of each sample as a template, 10 μL 2 × Fast sTaq PCR master mix (Servicebio, Wuhan China catG3304), 0.8 μL 10 μM forward primer, 0.8 μL 10 μM reverse primer, and 7.4 μL nuclease-free water. The primers for gene *PsEF1* (*Puccinia striiformis* elongation factor 1, accession number: KNE93481) are shown in Table S33. After being gently mixed and briefly centrifuged, the sqRT-PCR mixture was denatured at 95 °C for 2 min, then 30 cycles were amplified according to the setting of “denaturation at 95 °C for 10 s, annealing at 57 °C for 30 s, extension at 72 °C for 10 s” and finally extended at 72 °C for 10 min. Then, 5 μL of PCR product was loaded on 2.5% agarose gel containing 4S Green Plus Nucleic Acid Stain, and electrophoresis was performed using the setting “100 V, 45 min”. Grey-scale analysis of electrophoresis images was performed by ImageJ to indicate the relative expression level of *PsEF1* in each sample.

A 15 μL qRT-PCR mixture was prepared, which contained 1 μL cDNA of each sample as a template, 7.5 μL universal blue SYBR green qPCR master mix (Servicebio, Wuhan China G3326), 0.3 μL of 10 μM forward primer, 0.3 μL 10 μM reverse primer, and 5.9 μL nuclease-free water. The primers of reference gene *TaEF1α* (*Triticum aestivum* elongation factor 1 alpha, accession number: Q03033) and m^6^A regulator genes are shown in Table S33. After the qRT-PCR mixture was gently mixed and briefly centrifuged, it was placed in the CFX Connect fluorescent quantitative PCR detection system. The detection mode was adjusted to SYBR, and the denaturation was set at 95 °C for 30 s, and then 45 cycles were amplified according to the program of “denaturation at 95 °C for 15 s, annealing and extension at 60 °C for 30 s”. Finally, the melting curve was made at intervals of 0.5 °C for 5 s between 65 °C and 95 °C to determine the specificity of PCR amplification. Then, the amplified gene name, sample name, and operation repetition for each reaction well were set in CFX Maestro v2.2, and *TaEF1α* was set as the reference gene. The relative expression levels of each tested gene were calculated by the 2^−ΔΔCt^ method [63]. Each test was repeated five times.

## 5. Conclusions

In the differential gene analysis of RNA-seq, we found that among the significantly up-regulated 4100 genes (Figure 2C), 1010 wheat genes had similar expression patterns after stripe rust infection (Figure 2D, Table S7), including 18 genes annotated in the KEGG pathway as “Plant-pathogen interaction”, indicating that disease-resistance-related genes synergistically respond to resist the stripe rust assault. Among these eighteen genes, two are NBS-LRR disease resistance proteins (*TraesCS1A03G0059400*, *TraesCS7D03G0011300*), with *TraesCS1A03G0059400* annotated in UniProt as disease resistance protein RPM1 and *TraesCS7D03G0011300* as Rx N-terminal domain-containing protein. Two of them are pathogenesis-related protein 1 (*TraesCS5B03G1087600*, *TraesCS7D03G0450000*), and also, a significantly up-regulated *PR1* gene was found among the overall up-regulated genes (Figure 2E), indicating that the *TaPR1* gene is indeed an important gene in response to stripe rust, worthy of further research [37]. Six genes are annotated as “Calmodulin”, “Calcium-dependent protein kinase”, and “Cyclic nucleotide-gated channel”, indicating that calcium regulation plays a significant role in plant immune responses to affect the invasion of pathogens. Additionally, among the genes with the most significant decrease in m^6^A modification, we found that the most significantly down-regulated *TraesCS1A03G0058100* is annotated as an NBS-LRR disease-resistance protein. NBS-LRR genes play a crucial role in plant resistance against pathogens. This might be the first report of NBS-LRR genes showing hypo-methylation after infection in a *Pst*–wheat compatible interaction, suggesting that the m^6^A mechanism can directly regulate key genes in the plant immune pathway and affect the plant response to pathogens.

We observed how host wheat actively responds to stripe rust infection through the perspective of up-regulated genes suppressing the host wheat immune system for the successful invasion and colonization of *Pst*. We also turned our attention to the analysis of down-regulated genes. Among these genes, the two most significantly down-regulated are related to photosynthesis: *TraesCS1A03G0499300* (protein PAM68, chloroplast) and *TraesCS5D03G0016200LC* (photosystem I assembly protein Ycf4). When we further analyzed the overall significantly up-regulated and down-regulated genes in the enrichment of GO terms and KEGG pathways (Figure 3), we found that in the cellular component category of GO terms, there were strikingly multiple photosynthesis-related GO terms (Figure 3B, Table S10). Similarly, in the KEGG pathway enrichment, KO00196 (photosynthesis-antenna proteins) had the highest rich factor and the lowest *q*-value, with all 80 enriched genes being down-regulated (Figure 3D,E). This indicates that after compatible *Pst* infection, the photosynthesis-related genes in host wheat were significantly disrupted, and their regulatory levels were disturbed. The reduction in photosynthesis affects the accumulation of substances within the plant and the regulatory level of secondary biomass metabolism, as we also found that the regulatory levels of pathway genes in basic metabolism and secondary metabolism were reduced (for example, KO00710: carbon fixation in photosynthetic organisms; KO00630: glyoxylate and dicarboxylate metabolism) (Figure 3D,E). This may lead to a reduction in wheat resistance during the early stage of stripe rust infection. In the later stages of infection, the reduction in photosynthesis and substance accumulation forces the *Pst* to absorb nutrients from the host nearby leaf tissue, further expanding the invasion and colonization of *Pst*.

Additionally, this study also focused on the expression changes in m^6^A regulators in wheat after stripe rust infection. Among the 90 wheat m^6^A regulator genes summarized and annotated by Yue et al. (2019) [40], 28 m^6^A regulator genes were detected in this study (Figure 7A), indicating that these m^6^A regulator genes are closely associated with the response to stripe rust infection and are involved in m^6^A regulation. At the same time, the m^6^A modification levels of some m^6^A regulator genes themselves also showed significant changes after infection, such as hyper-methylation in *TaECT21* and *TaECT31* and hypo-methylation in *TaHAKAI1-A* and *TaHAKAI1-B*. This suggests that the regulation of m^6^A modification by m^6^A regulators is also under the control of the m^6^A mechanism, implying that the m^6^A mechanism is a precisely regulated biological process. The regulatory genes show time-point-dependent expressions; different genes have divergent dynamic response patterns, suggesting that the m^6^A methylation mechanism dynamically responds to the infection process. It is noticed that at 1 dpi, the expression levels of all tested m^6^A readers were lower. The observation is in accordance with the lower expression levels of m^6^A writers (Figure 7D–L), which is supported by previous results [40], indicating that most m^6^A writer genes also had lower expression levels in the early stages of stripe rust infection. Using the rust expression browser (http://www.rust-expression.com (accessed on 24 February 2024)) [64], we examined the expression levels of eight searchable m^6^A writer genes (*TaVIR-D*, *TaVIR-A*, *TaMTA-D*, *TaMTB-D*, *TaMTA-B*, *TaMTB-B*, *TaFIP37-2D*, *TaFIP37-2A*) in the wheat variety “Vuka” after infection with *Pst* isolates 87/66 at 0, 1, 2, 3, 5, 7, 9, and 11 dpi [65] (Figure S4) and found that most m^6^A writer genes also had lower expression levels in the early stages of stripe rust infection (2, 3 dpi).

Overall, we have produced an immense amount of data determining the genes undergoing m^6^A methylation for the first time in the wheat–*Pst* pathosystem. There is no doubt that these data will facilitate further in-depth analyses in system biology.

## Figures and Tables

**Table 1:** List of 24 annotations for 35 genes in the GO term related to “immune system process” using RNA-Seq data.

Annotations/Description/Gene Symbol	Gene ID	Regulation
UDP-glucose: glycoprotein glucosyltransferase (UGGT)	*TraesCS1A03G0409400* *TraesCS1D03G0377700*	Up
Lectin receptor kinase (LECRK91)	*TraesCS1B03G1249300* *TraesCS1D03G1014400*	Up
Receptor-like protein kinase, putative, expressed (CRK6)	*TraesCS2B03G0583500*	Up
GRAM domain family protein (VAD1)	*TraesCS2B03G0620400*	Up
Cyclic nucleotide-gated channel (CNGC4)	*TraesCS3A03G0774300*	Up
Calreticulin (CRT3)	*TraesCS3A03G0936700*	Up
Pathogenesis-related protein PR-4 (PR4A, PR4B)	*TraesCS3A03G1217600* *TraesCS3B03G1460900* *TraesCS3D03G1160600*	Up
Glutamate decarboxylase (SPL)	*TraesCS3B03G0229400* *TraesCS3D03G0168800*	Up
MACPF domain protein (CAD1)	*TraesCS3D03G0628000*	Up
Pheophorbide a oxygenase, chloroplastic (PAO)	*TraesCS4A03G1017200* *TraesCS4B03G0809700*	Up
Receptor protein kinase, putative (RLK7)	*TraesCS5A03G0031900*	Up
Cysteine protease (CCP2)	*TraesCS5B03G0553900*	Up
Aminotransferase-like protein (OAT)	*TraesCS5D03G0861400*	Up
Pleiotropic drug resistance ABC transporter (ABCG48)	*TraesCS5D03G0992800*	Up
E3 ubiquitin-protein ligase (ATL6)	*TraesCS6A03G0290200*	Up
Alcohol dehydrogenase, putative (CAD2)	*TraesCS6A03G0429300* *TraesCS6B03G0520600* *TraesCS6D03G0363400*	Up
Allene oxide cyclase (AOC)	*TraesCS6B03G1030800* *TraesCS6D03G0731500*	Up
BZIP transcription factor protein (BZIP50)	*TraesCS7A03G0964400*	Up
MACPF domain-containing protein (MACPF)	*TraesCS7B03G0282300*	Up
WRKY transcription factor (WRKY28)	*TraesCS7D03G1174900*	Up
GTPase Der (FZL)	*TraesCS1A03G0630100*	Down
Apolipoprotein D (CHL)	*TraesCS2D03G1058400*	Down
NAD(P)H-quinone oxidoreductase subunit N (ndhN)	*TraesCS3A03G0952700* *TraesCS3B03G1089400* *TraesCS3D03G0886900*	Down
Telomere-associated protein (RIF1)	*TraesCS7A03G0315600*	Down

**Table 2:** List of 18 annotations for 80 genes in the KEGG “plant-pathogen interaction pathway” using RNA-Seq data.

Annotations/Description/Gene Symbol	Gene ID	Regulation
Glycerol kinase (GLPK)	*TraesCS2B03G1367900*, *TraesCS2D03G1154600*	Up
Cysteine proteinase (CCP1)	*TraesCS2A03G0676600*, *TraesCS2D03G0630100*, *TraesCS5A03G0791200*, *TraesCS5B03G0823900*, *TraesCS5D03G0747200*	Up
Calmodulin, putative (CML27, CML37, CML4)	*TraesCS1B03G1255000*, *TraesCS1B03G1255400*, *TraesCS5A03G0918000*	Up
Heat shock protein 90 (HSP81-1)	*TraesCS7A03G0555500*, *TraesCS7D03G0537900*	Up
Cyclic nucleotide-gated channel (CNGC2)	*TraesCS5D03G0900900*	Up
Chaperone protein htpG family protein (HSP90-5, HSP90)	*TraesCS5B03G0646100*, *TraesCS5D03G0595900*, *TraesCS7A03G1288100*, *TraesCS7B03G1204400*, *TraesCS7D03G1221800*	Up
Calcium-dependent protein kinase (CPK15, CPK9, CPK10, CPK8, CPK4)	*TraesCS1D03G0995500*, *TraesCS4A03G0728000*, *TraesCS5A03G1009700*, *TraesCS5B03G1055900*, *TraesCS5D03G0955400*, *TraesCS5B03G1159600*, *TraesCS5D03G1051900*, *TraesCS6A03G0203200*, *TraesCS6B03G0271200*	Up
WRKY transcription factor (WRKY24)	*TraesCS1A03G0172500*, *TraesCS1A03G0743700*, *TraesCS1B03G0235500*, *TraesCS1B03G0848500*, *TraesCS1D03G0166900*, *TraesCS1D03G0711800*	Up
Kinase family protein (PTI13)	*TraesCS2D03G0794500*	Up
Respiratory burst oxidase (RBOHA, RBOHC, RBOHE)	*TraesCS3A03G0705100*, *TraesCS4B03G0848700*, *TraesCS5A03G1171300*, *TraesCS5D03G0694900*, *TraesCS5B03G0560500*, *TraesCS5D03G0519000*, *TraesCS6D03G0366700*	Up
Calcium-binding protein (CML19, CML27, CML36, CML5, CML21, CML8, CML25)	*TraesCS3B03G1378700*, *TraesCS4B03G0485600*, *TraesCS5A03G0382400LC*, *TraesCS5B03G0150400*, *TraesCS5B03G0370400*, *TraesCS5D03G0789100*, *TraesCS7B03G1109900*, *TraesCS7B03G1175200*, *TraesCS7D03G1164400*	Up
Pathogenesis-related protein 1-1 (PRMS)	*TraesCS5A03G0484700*, *TraesCS5A03G1037300*, *TraesCS5A03G1037400*, *TraesCS5B03G0483900*, *TraesCS5B03G1087600*, *TraesCS5D03G0980700*, *TraesCS5D03G0980800*, *TraesCS7D03G0362800*, *TraesCS7D03G0450000*	Up
RPM1-interacting protein 4 (RIN4)	*TraesCS7A03G0593800*	Up
NBS-LRR disease resistance protein, putative, expressed (RPM1, RPP13L4, PIK-2, PIK6-NP)	*TraesCS1A03G0059400*, *TraesCS1D03G0032300*, *TraesCS2D03G0030700*, *TraesCS2D03G1070500*, *TraesCS3D03G0966800*, *TraesCSU03G0241100*, *TraesCS1D03G0036500*, *TraesCS7B03G0033000*, *TraesCS7D03G0011300*	Up
Disease resistance protein (NBS-LRR class) (RPS2)	*TraesCS2A03G0930000*, *TraesCS2B03G1021500*, *TraesCS2D03G0868500*	Up
3-ketoacyl-CoA synthase (FDH, KCS20, CUT1, KCS11)	*TraesCS4A03G0012800*, *TraesCS4A03G0046100*, *TraesCS4B03G0728500*, *TraesCSU03G0063000LC*, *TraesCS7D03G0827800*	Up
Mitogen-activated protein kinase (MPK5)	*TraesCS4A03G0218300*	Up
Elongation factor Tu (TUFA)	*TraesCS6B03G0694200*, *TraesCS6D03G0480200*	Down

**Table 3:** List of 48 genes that exhibit a significant differentiation in both m^6^A-modified peaks and transcript abundance in *Pst*-infected wheat leaves in comparison to CK.

Gene ID, Strand ^a^	Description ^b^	Pattern ^c^	Peak Annotation ^d^	Peak Start ^e^	Peak End ^f^	IP_ L_2_FC ^g^	In_ L_2_FC ^h^
*TraesCS6B03G0561000*, +	Cytochrome P450 family protein	Hyper-up	5′, start, CDS	284010639	284010814	5.01	1.64
*TraesCS1D03G0829400*, −	Chaperone DnaJ	Hyper-up	stop, 3′	440701051	440701101	4.64	4.02
*TraesCS2B03G0935100*, −	Malate synthase	Hyper-up	start, CDS	526103000	526103125	4.48	4.57
*TraesCS5B03G0999600*, +	Chitinase	Hyper-up	start, CDS	583600714	583600764	4.50	3.12
*TraesCS7A03G1159700*, +	Ferredoxin--NADP reductase 2	Hyper-up	start, CDS	676526476	676526576	4.23	2.61
*TraesCS3A03G0941700*, −	Tropinone reductase-like protein	Hyper-up	5′, start, CDS	647714736	647714861	4.87	3.22
*TraesCS4D03G0669200*, −	DUF1997 family protein	Hyper-up	start, CDS	453670930	453670980	4.48	3.28
*TraesCS5A03G0935200*, −	Cysteine protease, putative	Hyper-up	5′, start, CDS	588519570	588519733	6.03	5.66
*TraesCS3B03G0133200*, −	D-Ala-D/L-Ala epimerase	Hyper-up	start, CDS	39875470	39875545	5.02	3.99
*TraesCS5B03G0752100*, −	Serine aminopeptidase S33	Hyper-up	5′, start, CDS	479815861	479816011	4.64	4.80
*TraesCS1D03G0951700*, +	GDP-mannose-3’,5’-epimerase	Hyper-up	5′	474501754	474501829	4.19	3.12
*TraesCS2D03G0725900*, +	Kinesin-like protein	Hyper-up	stop, 3′	407828185	407828373	6.57	3.53
*TraesCS3D03G0726600*, +	3-hydroxyacyl-CoA dehydrogenase	Hyper-up	stop, 3′	434958844	434958919	4.51	1.83
*TraesCS4A03G0035700*, −	plant/protein	Hyper-up	start, CDS	13580848	13580923	4.77	1.69
*TraesCS5D03G0513000*, +	Jasmonate zim-domain protein	Hyper-up	start, CDS	331598315	331598415	4.73	2.48
*TraesCS2A03G0387200*, +	vacuolar sorting-associated protein	Hyper-up	CDS	153236044	153236119	4.14	2.20
*TraesCS3B03G0090100*, +	Nuclease S1	Hyper-up	5′, start, CDS	24977420	24977620	4.13	2.69
*TraesCS1B03G0217600*, +	AAA+ ATPase	Hyper-up	start, CDS	75657827	75657977	4.38	1.63
*TraesCS2B03G1234100*, −	Amine oxidase family protein	Hyper-up	5′	695509349	695509524	4.68	1.63
*TraesCS2D03G0901800*, −	SPX domain-containing protein	Hyper-up	stop, 3′	509963790	509963915	4.27	2.57
*TraesCS2D03G0880500*, −	Aldehyde dehydrogenase	Hyper-up	stop, 3′	493975195	493975320	4.19	2.57
*TraesCS1A03G0652200*, +	Pyruvate phosphate dikinase	Hyper-up	stop, 3′	446585467	446585567	4.43	4.06
*TraesCS7D03G0606900*, +	Peptidylprolyl isomerase	Hyper-down	start, CDS	254192941	254192991	3.17	−1.75
*TraesCS3B03G1139000*, +	Alpha/beta hydrolase	Hyper-down	CDS	720605293	720605423	2.39	−1.57
*TraesCS3B03G1515300LC*, +	P-loop NTPase	Hypo-up	CDS, stop	847160574	847160674	−1.53	1.80
*TraesCS7D03G0910600*, +	ABC transporter	Hypo-up	start, CDS	502724870	502724945	−3.19	2.70
*TraesCS3A03G0325200*, +	GDSL esterase/lipase	Hypo-up	CDS, stop	131460529	131460623	−3.15	1.80
*TraesCS3A03G0354000*, +	Cytochrome P450, putative	Hypo-up	CDS	155007091	155007221	−1.50	1.98
*TraesCS1D03G0777100*, +	Cysteine protease family protein	Hypo-up	5′, start	421851652	421851802	−2.47	3.29
*TraesCS1A03G0652400*, −	Pyruvate phosphate dikinase	Hypo-up	start, CDS	447084657	447084809	−1.59	1.71
*TraesCS6B03G0574100*, −	CASP-like protein	Hypo-up	5′, start, CDS	296054675	296054825	−2.69	1.97
*TraesCS5A03G0517600*, −	Homeobox protein, putative	Hypo-up	5′	404317960	404318085	−2.37	2.24
*TraesCS1B03G0741000*, +	Pyruvate phosphate dikinase	Hypo-up	5′, start	471557711	471557786	−1.85	2.48
*TraesCS1B03G0574200*, −	Glutathione S-transferase	Hypo-up	5′, start, CDS	355708499	355708549	−2.56	1.52
*TraesCS1D03G0954900*, +	Chlorophyll *a*/*b* binding protein	Hypo-down	5′, start, CDS	475689826	475689876	−2.17	−3.10
*TraesCS2D03G0959100*, −	Cold-responsive proteinWCOR15	Hypo-down	5′, start, CDS	540101549	540101599	−3.47	−2.90
*TraesCS5D03G1027900*, −	Chlorophyll *a*/*b* binding protein	Hypo-down	5′, start, CDS	511061211	511061286	−1.67	−3.89
*TraesCS1D03G0729600*, −	Chlorophyll *a*/*b* binding protein	Hypo-down	5′, start, CDS	405463983	405464083	−2.37	−3.47
*TraesCS1B03G1053300*, +	Chlorophyll *a*/*b* binding protein	Hypo-down	start, CDS	629357113	629357163	−3.85	−3.69
*TraesCS6A03G0217100*, +	Chlorophyll *a*/*b* binding protein	Hypo-down	start, CDS	64267362	64267412	−2.64	−3.37
*TraesCS5B03G1135900*, −	Chlorophyll *a*/*b* binding protein	Hypo-down	5′, start, CDS	638614728	638614778	−2.47	−3.18
*TraesCS1B03G1053200*, +	Chlorophyll *a*/*b* binding protein	Hypo-down	start, CDS	629262631	629262681	−3.97	−3.86
*TraesCS5D03G1027800*, −	Chlorophyll *a*/*b* binding protein	Hypo-down	5′, start, CDS	511058147	511058297	−3.14	−3.25
*TraesCS1A03G0988100*, +	Chlorophyll *a*/*b* binding protein	Hypo-down	5′, start, CDS	569868615	569868715	−4.36	−3.34
*TraesCS1D03G0873500*, −	Chlorophyll *a*/*b* binding protein	Hypo-down	CDS	454772861	454772911	−4.66	−3.19
*TraesCS1A03G0761900*, −	Chlorophyll *a*/*b* binding protein	Hypo-down	5′, start, CDS	500100318	500100462	−5.01	−3.65
*TraesCS1B03G0871200*, −	Chlorophyll *a*/*b* binding protein	Hypo-down	5′, start	549194399	549194449	−3.22	−3.48
*TraesCS2D03G0286600LC*, −	RING/U-box superfamily protein	Hypo-down	start, CDS	82356920	82356989	−2.24	−3.05

^a^ The numbers and letters in bold in wheat gene names represent the chromosome where the gene is located. Wheat genes come from the sense strand (+) or antisense strand (−) of DNA; ^b^ gene descriptions come from WheatOmics (http://wheatomics.sdau.edu.cn/ (accessed on 13 February 2024)) or UniProtKB (https://www.uniprot.org/uniprotkb (accessed on 13 February 2024)); ^c^ The pattern indicates whether the m^6^A-modified peak of the differential gene is hyper-methylated or hypo-methylated and whether the transcript is up-regulated or down-regulated. The background colors are pink for hyper-up, purple for hyper-down, yellow for hypo-up, and green for hypo-down. ^d^ Distribution of m^6^A-modified peaks on different gene functional elements. “5′ “is 5′ untranslated region (UTR), “start” is start codon, “CDS” is coding sequence, “stop” is stop codon, and “3′ “is 3′ UTR. ^e^ The starting position of the m^6^A-modified peak in the chromosome; ^f^ the termination position of the m^6^A-modified peak in the chromosome. ^g^ In MeRIP-seq (IP) analysis, the fold change (FC) of m^6^A-modified peaks in infected samples relative to the CK is calculated and then subjected to log_2_ transformation (L2); ^h^ in RNA-seq (Input, In) analysis, the fold change (FC) of transcripts in infected samples relative to the CK is calculated and then subjected to log_2_ transformation (L2). The colors distinguish the genes showing differential patterns of regulation: pink for hyper-up, purple for hyper-down, yellow for hypo-up, and green for hypo-down, the same color scheme was used in Figure 6C.

## Data Availability

The datasets presented in this study can be found in online repositories. The name of the repository and accession number can be found below: National Center for Biotechnology Information (NCBI) BioProject, PRJNA1072674.

## Supplementary Materials

The following supporting information can be downloaded at https://www.mdpi.com/article/10.3390/plants13070982/s1, Figure S1. Uncropped agarose gel (2.5%) electrophoresis pattern of semi-quantitative reverse transcription PCR (sqRT-PCR) product for *PsEF1*; Figure S2. Distribution and coverage of m^6^A peaks from CK sample (A) and infected sample (B) on wheat chromosome; Figure S3. Number of m^6^A peaks distributed within 5′UTR (untranslated region) (A), start codon (B), CDS (coding sequence) (C), stop codon (D), and 3′UTR (E) of genes in CK sample and infected sample; Figure S4. The heatmap visualization of the expression levels of m^6^A writer genes in the wheat variety “Vuka” when infected with *Pst* isolate 87/66, detected at 0, 1, 2, 3, 5, 7, 9, and 11 dpi, as logged in the rust expression browser. Table S1. Data filtering statistics of RNA-seq; Table S2. Base information statistics of RNA-seq; Table S3. Ribosomal RNA mapping statistics of RNA-seq; Table S4. Reference genome mapping statistics of RNA-seq; Table S5. All gene annotations of RNA-seq; Table S6. Annotation of all genes with significant differential expression in RNA-seq; Table S7. Heatmap clustering of differentially expressed genes (DEGs); Table S8. Gene Ontology (GO) terms enrichment in biological processes of differentially expressed genes (DEGs); Table S9. Gene Ontology (GO) terms enrichment in molecular function of differentially expressed genes (DEGs); Table S10. Gene Ontology (GO) term enrichment in cellular component of differentially expressed genes (DEGs); Table S11. Level 2 Gene Ontology (GO) terms of differentially expressed genes (DEGs); Table S12. KEGG enrichment analysis of differentially expressed genes (DEGs); Table S13. Data filtering statistics of MeRIP-seq; Table S14. Base information statistics of MeRIP-seq; Table S15. Ribosomal RNA mapping and filtering statistics of MeRIP-seq; Table S16. Reference genome mapping statistics of MeRIP-seq; Table S17. CK sample peak identification of MeRIP-seq; Table S18. Infected sample peak identification of MeRIP-seq; Table S19. CK sample peak-related gene annotation of MeRIP-seq; Table S20. Infected sample peak-related gene annotation of MeRIP-seq; Table S21. Statistics of peak number in each group; Table S22. *Pst*-induced methylation sites and associated genes in infected sample motif; Table S23. All peak annotations of MeRIP-seq after merging peaks; Table S24. Annotation of differentially m^6^A-modified peaks; Table S25. Gene Ontology (GO) terms enrichment in biological processes of differentially m^6^A-modified peaks (DMPs); Table S26. Gene Ontology (GO) terms enrichment in cellular component of differentially m^6^A-modified peaks (DMPs); Table S27. Gene Ontology (GO) terms enrichment in molecular function of differentially m^6^A-modified peaks (DMPs); Table S28. Level 2 Gene Ontology (GO) terms of differentially m^6^A-modified peaks (DMPs); Table S29. KEGG enrichment analysis of differentially m^6^A-modified peaks (DMPs); Table S30. Joint statistics on fold changes of m^6^A-modified peak-related genes and transcripts in infected leaves in comparison to CK; Table S31. Significant fold changes of both m^6^A-modified peak-related genes and transcripts in infected leaves in comparison to CK; Table S32. Accession numbers, amino acid sequences, and name codes of m^6^A writers, erasers, and readers of *Triticum aestivum*; Table S33. The primers used in this work.
