# Supplementary material for: Transcriptome-Wide N6-Methyladenosine (m6A) Methylation Analyses in a Compatible Wheat–Puccinia striiformis f. sp. tritici Interaction
Source: Plants (Basel). 2024 Mar 29;13(7):982. doi: 10.3390/plants13070982 (PMC11013425; doi:10.3390/plants13070982)
Supplement: Supplementary file 1 [file plants-13-00982-s001.zip › Supplementary Figures Cerav et al., 2024.pdf]

Supplementary Figures

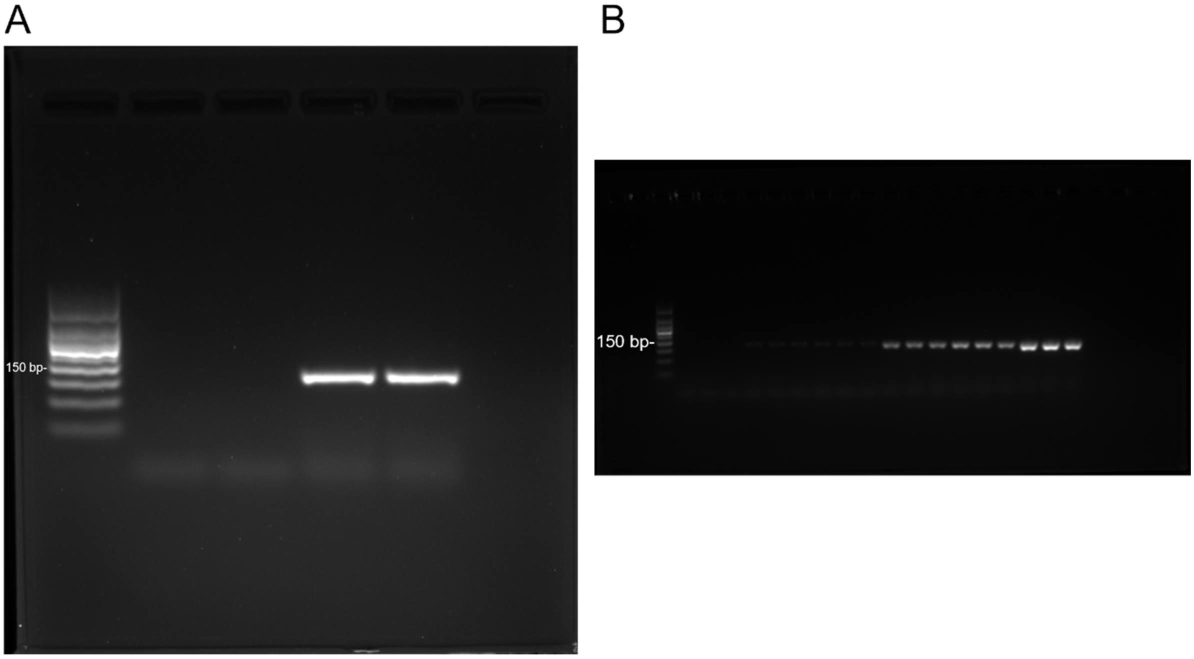

Supplementary Figure S1. Uncropped agarose gel (2.5%) electrophoresis pattern of semi-quantitative reverse transcription PCR (sqRT-PCR) product for *PsEF1*. (**A**) Agarose electrophoresis image of Figure 1D without cropping. (**B**) Agarose electrophoresis image of Figure 7B without cropping.

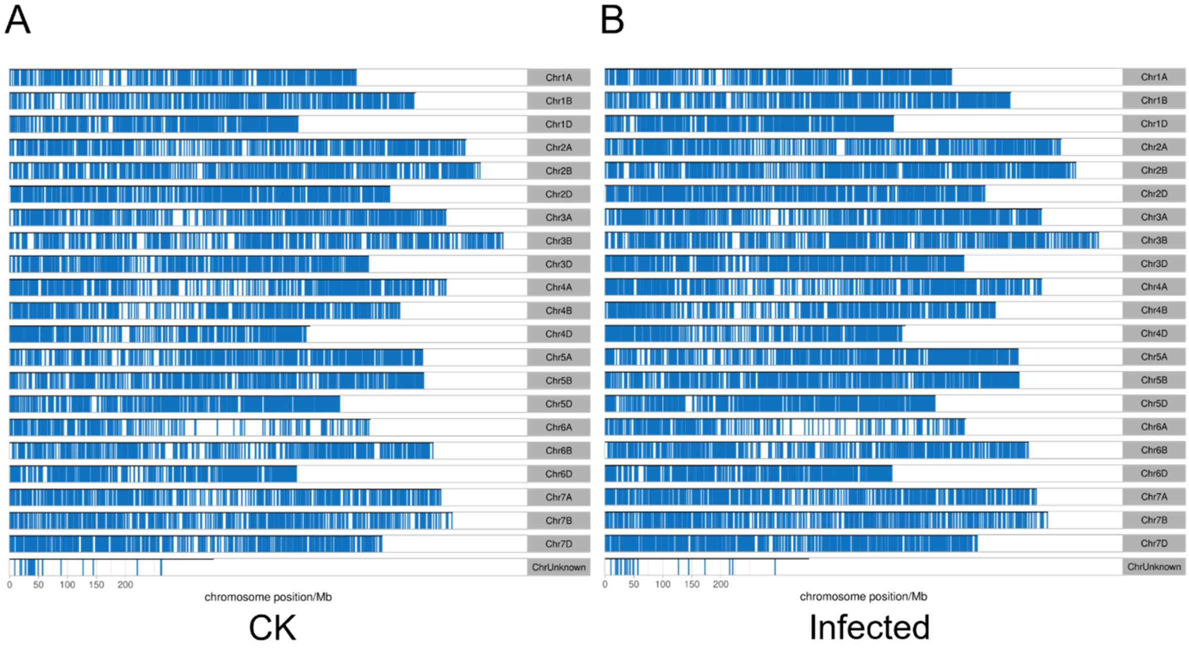

Supplementary Figure S2. Distribution and coverage of m^{6}A peaks from CK sample (**A**) and infected sample (**B**) on wheat chromosome.

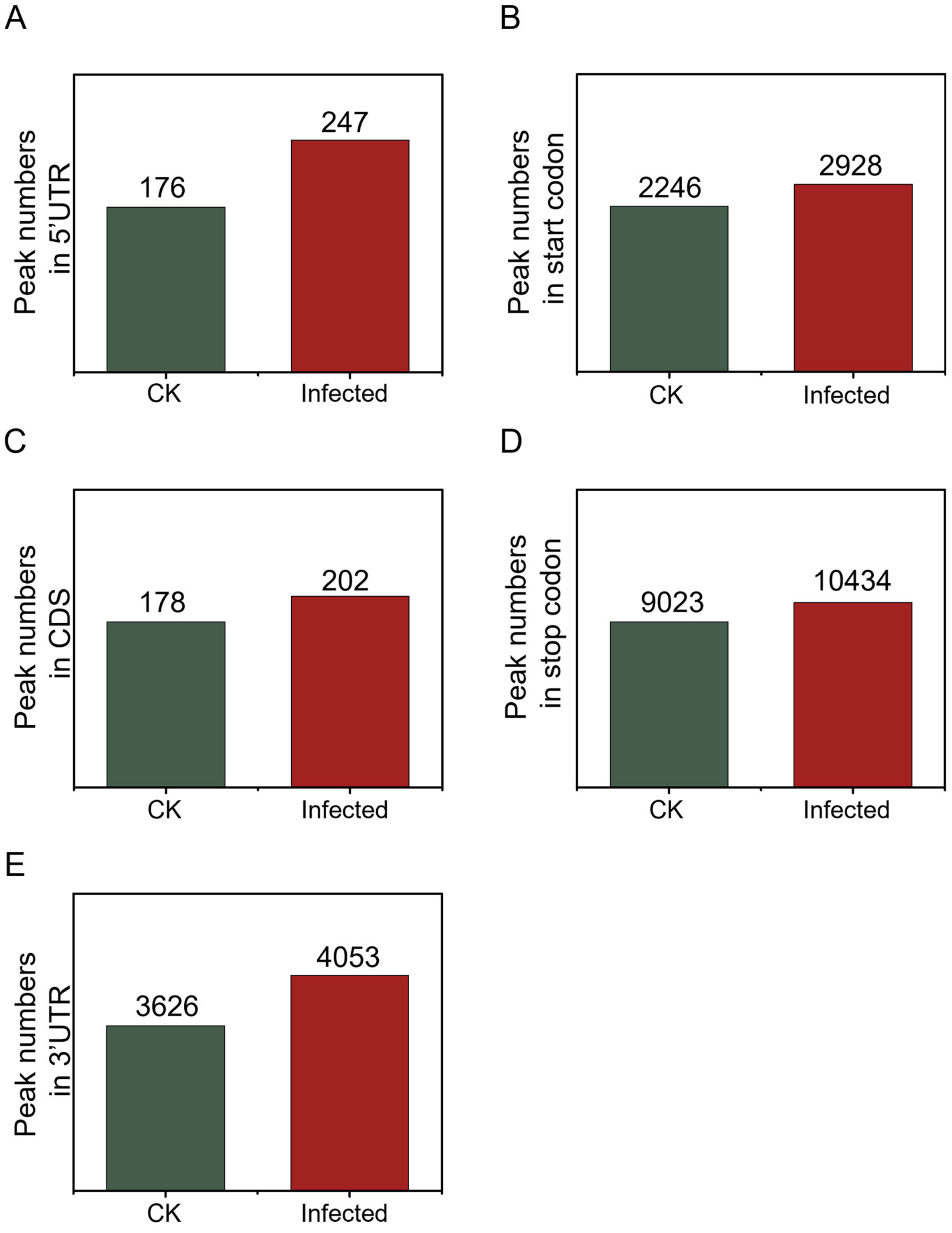

Supplementary Figure S3. Number of m^6A peaks distributed within 5'UTR (untranslated region) (**A**), start codon (**B**), CDS (coding sequence) (**C**), stop codon (**D**) and 3'UTR (**E**) of genes in CK sample and infected sample.

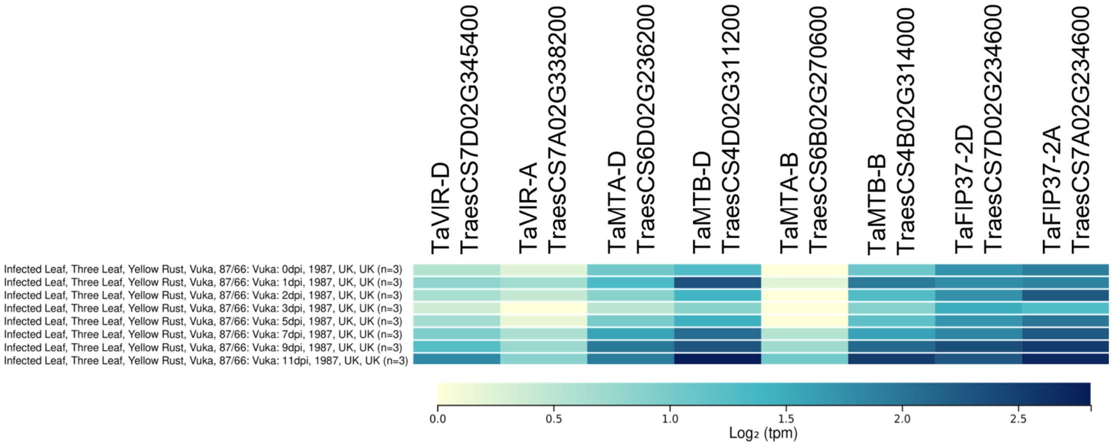

Supplementary Figure S4. The heatmap visualization of the expression levels of m6A writer genes in the wheat variety 'Vuka' when infected with Pst isolate 87/66, detected at 0, 1, 2, 3, 5, 7, 9, and 11 dpi, as logged in the Rust Expression Browser.
